# Development of Matrix Metalloproteinase-2 Inhibitors for Cardioprotection

**DOI:** 10.3389/fphar.2018.00296

**Published:** 2018-04-05

**Authors:** Péter Bencsik, Krisztina Kupai, Anikó Görbe, Éva Kenyeres, Zoltán V. Varga, János Pálóczi, Renáta Gáspár, László Kovács, Lutz Weber, Ferenc Takács, István Hajdú, Gabriella Fabó, Sándor Cseh, László Barna, Tamás Csont, Csaba Csonka, György Dormán, Péter Ferdinandy

**Affiliations:** ^1^Cardiovascular Research Group, Department of Biochemistry, Faculty of Medicine, University of Szeged, Szeged, Hungary; ^2^Pharmahungary Group, Szeged, Hungary; ^3^Department of Biochemistry, Faculty of Medicine, University of Szeged, Szeged, Hungary; ^4^Department of Pharmacology and Pharmacotherapy, Faculty of Medicine, Semmelweis University, Budapest, Hungary; ^5^Infarmatik, Budapest, Hungary; ^6^OntoChem GmbH, Halle (Saale), Germany; ^7^Targetex Biosciences, Dunakeszi, Hungary; ^8^Research Centre for Natural Sciences, Institute of Enzymology, Hungarian Academy of Sciences, Budapest, Hungary; ^9^Microscopy Center at IEM HAS, Institute of Experimental Medicine, Hungarian Academy of Sciences, Budapest, Hungary

**Keywords:** matrix metalloproteinase, MMP-2 inhibitor, heart, ischemia/reperfusion injury, cardioprotection, lead candidate

## Abstract

The objective of our present study is to develop novel inhibitors for MMP-2 for acute cardioprotection. In a series of pilot studies, novel substituted carboxylic acid derivatives were synthesized based on imidazole and thiazole scaffolds and then tested in a screeening cascade for MMP inhibition. We found that the MMP-inhibiting effects of imidazole and thiazole carboxylic acid-based compounds are superior in efficacy in comparison to the conventional hydroxamic acid derivatives of the same molecules. Based on these results, a 568-membered focused library of imidazole and thiazole compounds was generated *in silico* and then the library members were docked to the 3D model of MMP-2 followed by an *in vitro* medium throughput screening (MTS) based on a fluorescent assay employing MMP-2 catalytic domain. Altogether 45 compounds showed a docking score of >70, from which 30 compounds were successfully synthesized. Based on the MMP-2 inhibitory tests using gelatin zymography, 7 compounds were then selected and tested in neonatal rat cardiac myocytes subjected to simulated I/R injury. Six compounds showed significant cardio-cytoprotecion and the most effective compound (MMPI-1154) significantly decreased infarct size when applied at 1 μM in an *ex vivo* model for acute myocardial infarction. This is the first demonstration that imidazole and thiazole carboxylic acid-based compounds are more efficacious MMP-2 inhibitor than their hydroxamic acid derivatives. MMPI-1154 is a promising novel cardio-cytoprotective imidazole-carboxylic acid MMP-2 inhibitor lead candidate for the treatment of acute myocardial infarction.

## Introduction

Coronary heart disease (CHD) is the number one cause of death globally (Alwan et al., 2010). Recent data show that almost 18 million people died from CVDs in 2015, of which an estimated 7.4 million were due to coronary heart disease (Roth et al., 2017; WHO, 2017). The discovery of endogenous cardioprotective mechanisms (Ischemic pre-, post-, and remote pre- and perconditioning) has allowed for the exploration of several molecular processes of cell injury and survival mechanisms during ischemia/reperfusion (I/R) (Ferdinandy et al., 2014). However, in spite of numerous promising preclinical attempts aiming pharmacological triggering these cardioprotective mechanisms, the dilemma of translation of the results into clinical practice has remained unsolved due to the presence of several additional factors including cardiovascular co-morbidities (e.g., hyperlipidemia or diabetes mellitus) (Ferdinandy et al., 2014). Thus, to improve clinical outcomes, novel therapeutic strategies against myocardial I/R injury are needed, which may preserve their protection even in the presence of cardiovascular co-morbidities (Hausenloy et al., 2017).

Matrix metalloproteinases (MMP) are zinc containing peptidases classified into several subtypes. The gelatinase-type MMP-2 occurs in the heart in physiological conditions and is synthesized by cardiomyocytes, fibroblasts, and endothelial cells (DeCoux et al., 2014). During I/R, MMP-2 is activated and released from the injured myocardium (Cheung et al., 2000), which may contribute to the degradation of contractile proteins (Wang et al., 2002; Sawicki et al., 2005; Sung et al., 2007; Ali et al., 2010), thereby leading to myocardial dysfunction, and in the long run, to heart failure. Furthermore, in patients with ST-elevation myocardial infarction (STEMI), a significant positive correlation has been shown between the circulating levels of MMP-2 measured before and 12 h after recanalization therapy, and infarct size as determined by cardiac MR (D'Annunzio et al., 2009). We have demonstrated that MMP-2 can be a promising biomarker for patients with coronary artery disease (Bencsik et al., 2015). We have previously also reported that pharmacological inhibition of MMP-2 in rats evoked cardioprotection that is equivalent to ischemic preconditioning (Giricz et al., 2006; Bencsik et al., 2010). Our work has also shown that although hyperlipidemia abolished the beneficial effect of ischemic preconditioning, cardioprotection in the presence of hyperlipidemia was preserved during pharmacological inhibition of MMP-2 (Giricz et al., 2006). We can thus conclude that MMP-2 inhibition is a promosing drug target since it works in the presence of a significant cardiovascular co-morbidity, namely hyperlipidemia (see for reviews Andreadou et al., 2017).

To date, several MMP inhibitors have been identified, including hydroxamates, thiols, carbamoylphosphonates, hydroxyureas, hydrazines, β-lactam, squaric acids, and nitrogenous ligands (Durrant et al., 2011). Most of these consist of a metal-coordinating function, called a zinc-binding group (ZBG), which binds to the catalytic zinc ion of the MMPs. Despite the promising features of these potent MMP inhibitor compounds, only one compound has been approved for clinical use by the U.S. Food and Drug Administration Authority, which is Periostat® (doxycycline hyclate), for the treatment of periodontitis (Dormán et al., 2010). In spite of much preclinical evidence about the involvement of MMP-2 in acute myocardial infarction (AMI), surprisingly, only one failed clinical trial was conducted by the administration of a non-selective, hydroxamate type MMP inhibitor, PG-116800, in a relatively high dose (400 mg/day) for 90 days for AMI patients (Hudson et al., 2006).

Consequent research has been focused on the design of selective compounds that can distinguish between different members of the MMP family, thereby exploiting zinc-binding groups other than the hydroxamate group (Fisher and Mobashery, 2006). In addition, we have recently shown that there is no need for complete inhibition of MMP-2 to achieve cardioprotection since a moderate (~20–25%) inhibition of MMP-2 activity was sufficient to reduce infarct size in normo- and hyperlipidemic isolated rat hearts (Giricz et al., 2006) and also in an *in vivo* rat model of AMI (Bencsik et al., 2014).

Consequently, our aims were to develop novel MMP-2 inhibitors with potent anti-ischemic efficacy and moderate MMP-2 selectivity among the MMP-subtypes. Preclinical studies with MMPI's revealed a severe adverse side-effect frequently, referred to as musculoskeletal syndrome. This is primarily due to MMP-1 inhibition (which is considered an anti-target within the MMPs). Selectivity against MMP-1 may be important to avoid such side effects of MMP inhibitors (Papp et al., 2007).

**Figure 1:**
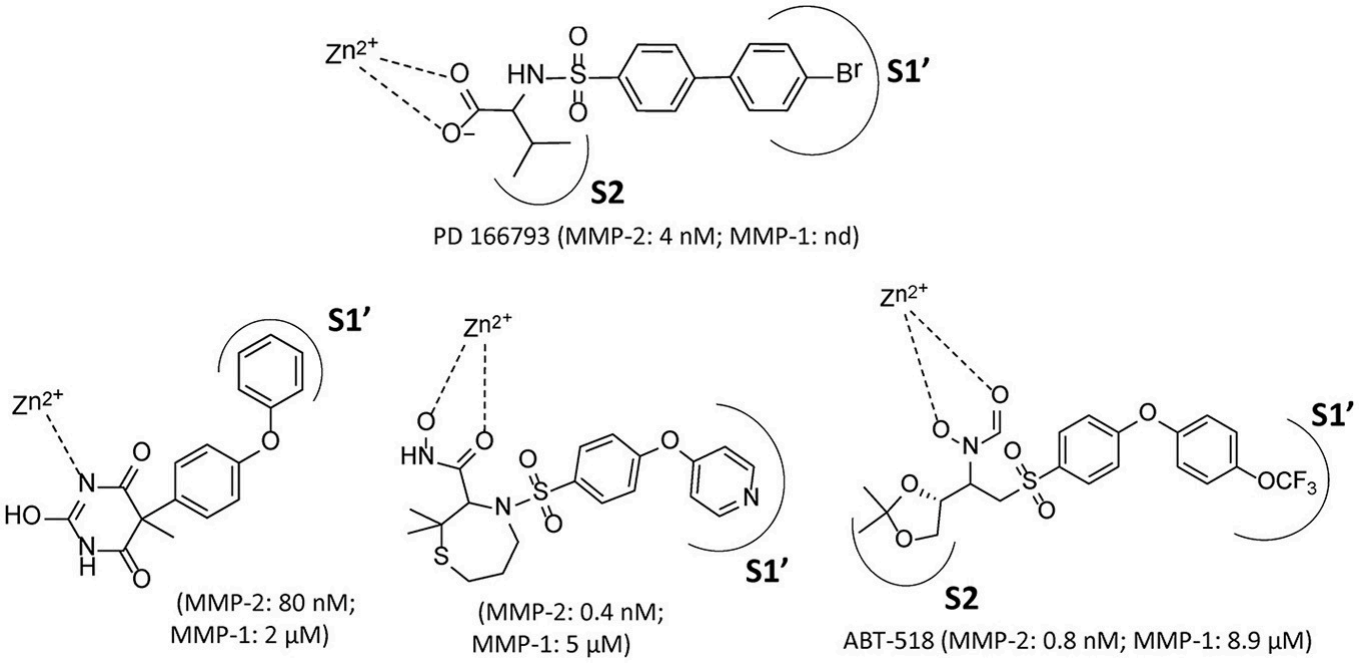
Selected MMP-2 inhibitors and their subpocket occupation leading to selectivity against MMP-1 (Corresponding IC50 values are shown).

The significant differences in the structural features of the sub-pockets of the binding/active sites allow for easy differentiation and selectivity of the MMP inhibitors. S1' and S2' pockets are responsible for the selectivity of the inhibitors and this can be taken into consideration in the design of selective inhibitors to tailor the occupation of the particular sub-pockets (Figure 1). In the case of MMP-2, the S1' pocket is mainly hydrophobic and relatively large, while in MMP-1 it is short and shallow. Increasing bulkiness at the S1' pocket could change the activity profile and allows for some selectivity over MMP-1. This trend was clearly observed in the case of substituted thiazepine MMP inhibitors (Almstead et al., 1999; Papp et al., 2007).

Therefore, we have designed a screening cascade to select potent MMP-2 inhibitors with cardioprotective effects.

## Materials and methods

### Experimental design-screening cascade

**Figure 2:**
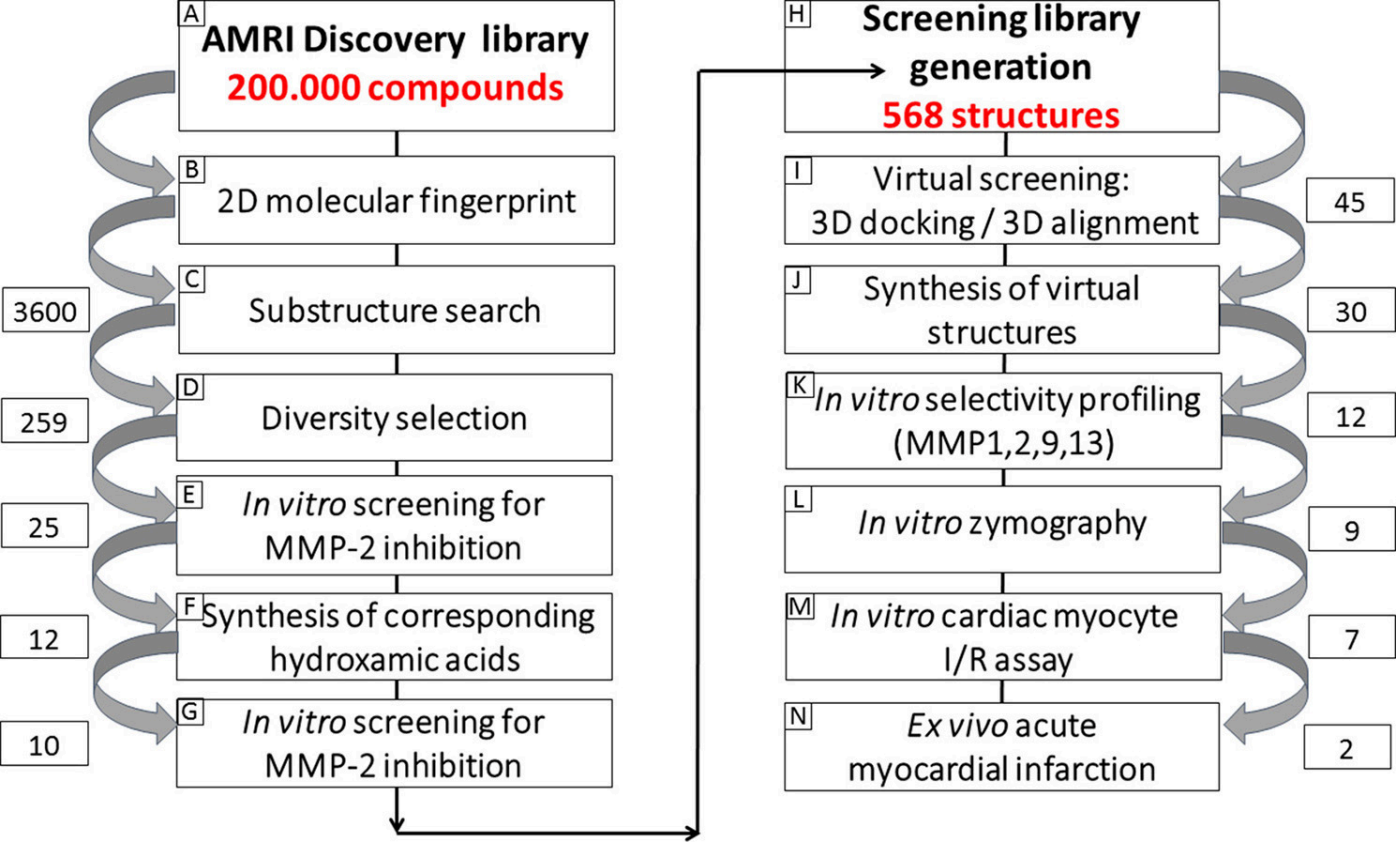
The screening cascade. Complex screening cascade to identify candidates that may reduce acute cardiac I/R injury via inhibition of MMP-2. **(A)** AMRI Chemical Library contains ~200,000 drug-like small molecules (<500 Da) as compound set. We intended to select zinc-binding motif holding molecules, similar to hydroxamic acids. **(B)** For 2D substructure and similarity search. **(C)** Selection of free acids from the AMRI's compound's collection. **(D)** Further focus to compounds holding various motifs around a central core, reflecting the typical MMP inhibitor architecture. **(E)** Selected acids screened in a fluorescent assay using a recombinant human MMP-2 catalytic fragment and a synthetic peptide substrate. **(F)** The synthesis of the thiazole and the isosteric imidazole carboxylic acids. **(G)** The hydroxamic acid pairs of the previously measured acids were tested. **(H)** The novel thiazole carboxylic acid chemotype was the starting point for further structure-based optimization. A 568-membered focused library was *in silico* generated around the AMRI library hits including their bioisosters and some simplified analog. **(I)** Docking studies: Genetic Optimization for Ligand Docking (GOLD) was used to build a 3D model based on the X-ray structure of human MMP-2 and MMP-9. **(J)** Thirty compounds were successfully synthesized for screening combining the *in silico* hits and the additional designed compounds. **(K)** *In vitro* MMP-2 activity was measured using a fluorometric assay. **(L)** Low throughput screening by gelatin zymography technique. **(M)** Cell viability experiments in isolated neonatal cadiac myocytes subjected to simulated ischemia/reperfusion injury. **(N)** Myocardial infarct size was measured after *ex vivo* global ischemia experiments on isolated rat hearts.

Our group applied a complex screening cascade to identify candidates that may reduce acute cardiac I/R injury via inhibition of MMP-2. During our complex screening protocol, virtual screening was combined with docking calculations followed by medium-throughput screening using MMP-2 catalytic domain. In the next stage, the inhibitory effect was confirmed on full length MMP-2 enzyme isolated from cardiac tissue. Finally, the cardioprotective effects of selected molecules were tested in neonatal cardiac myocytes that were subjected to simulated ischemia and reoxygenation as well as on an isolated rat heart model of AMI (Figure 2).

### Chemistry—MMP-2 inhibitor design

#### Design of selective MMP-2 inhibitors

We applied contemporary library design approaches based on the structural features of the known MMP-2 inhibitors (Figure 2). Our approach started from a diverse 200k compound library and the multi-step selection procedure consisted of a substructure search for binding motifs of MMP-2 inhibitors and diversity selection.

#### *In silico* chemisty approach

##### Chemical library

The Albany Molecular Research Inc. (AMRI; Albany, NY) Library contains ~200,000 drug-like small molecules (<500 Mwt) synthesized by solution phase parallel synthesis. The compound set contained ~300 medicinal chemistry relevant chemotypes with diverse substitution patterns. The library was succesfully involved in many exclusive drug discovery projects.

##### 2D chemoinformatics methods

According to the Similar Property Principle (Johnson and Maggiora, 1990), molecules that are structurally similar are likely to have similar properties. Applying simple 2D fingerprints is often the method of choice, particularly when numerous reference compounds and multimillion compound databases are available not only “because of its computational efficiency but also because of its demonstrated effectiveness in many comparative studies” (Willett, 2006; Baig et al., 2016). Most frequently the Tanimoto coefficient (Willett and Winterman, 1986) is used for measuring similarity, in spite of its marked size-dependency.

In practice, determining the similarity between known reference structures and each molecule in a database, followed by ranking the database molecules according to the similarities would lead to a potentially active compound set for *in vitro* screening. Similarly, reoccurring (privileged) structural motifs could also be identified and the compounds holding the motifs could represent another screening library.

For 2D substructure and similarity search, we applied standard chemical fingerprints as implemented into InstantJChem software (ChemAxon Ltd. Budapest) in which binary strings encode the presence or absence of substructures.

The physico-chemical parameters [Mwt, clogP, H-bond donors/acceptors,—Lipinski's Rule-5 (Lipinski et al., 2001); rotatable bonds, and topological polar surface area] were calculated by the calculation suit of InstantJChem (ChemAxon Ltd. Budapest).

##### 3D alignment methods

Novel 3D approaches consider not only the molecular topology, but also deal with 3D coordinates of both the active and the potential lead molecules for the similarity comparison and estimate 3D shape similarity (Kalászi et al., 2014).

A rough estimation of the binding behavior of the compounds is to assess their conformational flexibility and the overall statistical representation of such conformational properties would be presented as a 3D structure (ChemAxon Screen3D software) (ChemAxon, 2013).

In flexible alignment, the conformations are created “on-the-fly” during the alignment procedure. Flexible alignment methods, such as used in the present study, have the advantage of not requiring a pre-defined set of initial conformers to sample the conformational space of the molecules. During the alignment procedure we took specific atom-type information such as pharmacophore sites into account. This information would be capable of generating alignments where patterns (With similar binding character) are oriented in a similar fashion as occurs during the real binding to the active site. Therefore, it provides a more realistic picture of the potential bioactive similarity of the molecules.

##### 3D modeling approaches

For docking studies, Genetic Optimization for Ligand Docking (GOLD; version 4.0.1; Jones et al., 1997) was used to build a 3D molecule model based on the X-ray structure of human MMP-2. 1CK7 was the only full length 3D structure found in protein databases but it contained a mutation (E404A). On the other hand, the availability of the 3D structure of the collagenase-like 1-2 catalytic domain is sufficient for virtual screening targeting MMP-2 inhibition, thus 1HOV (NMR), and 1QIB (X-Ray) structures provided feasible alternatives.

Another option was 1EAK (X-Ray), which contains the collagenase-like 1-2 domain together with connecting collagen binding region (propeptides). Comparing the models 1EAK was found to be the particularly reliable for virtual screening even though it also contains the E404A mutation (Supplementary Figure 1). The propeptide regions could be removed without affecting the docking realiability.

The 3D structure of small molecules to be screened were optimized and protonated before docking. The pH was set as 7.2. For docking the standard Gold parameters were used as described in the actual User Guide (Centre, 2017).

The MMP-2 active site was defined containing all the atoms around a sphere with 19 Å radius. We have chosen Zn-ion coordination as octahedral. For all the small molecules 10 independent runs were conducted.

The 1EAK model was validated with three known MMP-2 inhibitors: SC-74020 (Supplementary Figure 2), PD 166793 (Figure 1), and ABT-518 (Figure 1).

### Synthetic methods

The hydroxamic acids (e.g., AMRI-101H, AMRI-102H, and AMRI-103H) were prepared from the corresponding acids using bromo-tris-pyrrolidino phosphoniumhexafluorophosphate (PyBrOP) and polymer supported hydroxybenzotriazole as activating agent before adding hydroxylamine hydrochloride and a base (see Supplementary Figure 3). The isolated yields were between 10 and 76%, while the purity was higher than 85%.

The synthesis of the thiazole and the isosteric imidazole carboxylic acids were carried out according to standard procedures and as described elsewhere (Ferdinandy et al., 2010).

In order to increase the solubility of the compounds, the benzene ring was replaced with pyridine in various analogs (MMPI-1248, MMPI-1260). Unfortunately, combination of the pyridine ring with the imidazolyl core was synthetically unsuccessful.

### *In vitro* pharmacological testing by MTS screening

*In vitro* MMP-2 activity was then measured, using a fluorometric assay in a 384 well format. Human MMP-2 catalytic domain (residues 110-221, 397-455) (Feng et al., 2000) was expressed in *E. coli* in form of inclusion bodies. The protein was refolded and then purified by means of Ni-NTA affinity and anion exchange chromatography. Inhibition assays were carried out in 50 mM Tris, 5 mM CaCl_2_, 300 mM NaCl, 20 μM ZnSO_4_, pH = 7.5 buffer. For inhibition studies the catalytic domain of the enzyme was pre-incubated with varying amount of inhibitor for 30 min. Then MMP substrate (Mca-Pro-Leu-Gly-Leu-Dpa-Ala-Arg-NH2) (Papp et al., 2007) was added at 3 μM final concentration. After 1 h incubation at 37°C the fluorescence was detected using a Wallac 1420 Victor2 microplate reader at 320 nm/405 nm Ex/Em wavelength. As an alternative substrate we also used 5-FAM-Pro-Leu-Gly-Leu-Dap(QXL™ 520)-Ala-Arg-NH_2_, where the fluorescence was detected at 485 nm/520 nm. For each inhibitor candidate, the percentage of inhibition was determined in duplicate experiments at six inhibitor concentrations, chosen to observe a 5–95% range of inhibition. For validation of the fluorometric assay, Ilomastat [N-[(2R)-2-(Hydroxamidocarbonylmethyl)-4-methylpentanoyl]-L-tryptophan Methylamide, (GM6001)], a non-selective MMP inhibitor, was used as a positive control inhibitor. The measured IC50 values varied between 0.3-1.0 nM which is in line with previous literature data (Galardy et al., 1994; Yamamoto et al., 1998).

### Gelatin zymography assay to screen the efficacy of MMP-inhibitiors

Gelatin zymography was performed as described previously (Kupai et al., 2010; Bencsik et al., 2017). MMP-2 was isolated from rat heart homogenates as follows: 50 μg protein/lane were loaded and separated by electrophoresis under non-reducing conditions on an 8% SDS-polyacrylamide gels copolymerized with 2 mg/ml gelatin from porcine skin (Sigma-Aldrich; St. Louis, MO). After electrophoresis, gels were washed in 2.5% Triton-X 100 with gentle agitation and then incubated for 20 h at 37°C in zymography development buffer (50 mM Tris-HCl, pH 7.5, containing 5 mM CaCl_2_, 200 mM NaCl) in the presence or abscence of the MMP inhibitor compounds. Zymographic gels were stained in a 0.05% Coomassie Brilliant Blue R-250 solution followed by destaining, and then zymograms were scanned. MMP activity was detected as a colorless transparent zone on a blue background and the clear bands in the gel were quantified by densitometry using the Quantity One software (Bio-Rad, Hercules, CA). The obtained density values were measured and percentage of inhibition values were then calculated.

### Cytoprotective effect of MMP inhibitor compounds in neonatal rat cardiac myocytes subjected to simulated ischemia/reperfusion (SI/R)

#### Simulated ischemia/reperfusion injury under hypoxic cinditions

**Figure 3:**
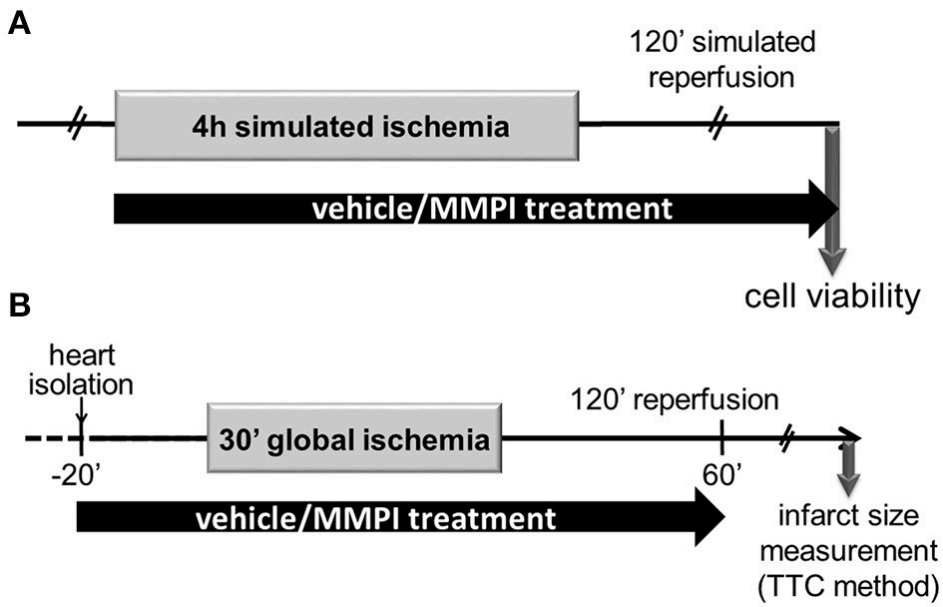
Experimental protocol for cell culture studies and for the *ex vivo* rat heart model of AMI. **(A)** Isolated neonatal rat cardiac myocytes were subjected to 4 h of simulated ischemia followed by 2 h of simulated reperfusion. At the end of the reperfusion, cell viability was determined by using calcein flurescence. **(B)** Isolated adult rat hearts were perfused according to Langendorff and a 30-min global, no-flow ischemia was applied after a 20 min equilibration period. Subsequently, 2 h reperfusion was applied and then infarct size was determined. The hearts were perfused with Krebs-Henseleit solution containing lead candidates or vehicle from 20 min prior to the global ischemia until the 60th min of reperfusion.

For our cell viability experiments, 3 day-old cardiomyocytes plated onto 24-well plates were tested under normoxic condition or subjected to simulated ischemia (SI). The normoxic cardiomyocytes were kept under normoxic conditions, i.e., the growth medium was changed to a normoxic solution (in mM: NaCl 125, KCl 5.4, NaH_2_PO_4_ 1.2, MgCl_2_ 0.5, HEPES 20, glucose 15, taurine 5, CaCl_2_ 1, creatine 2.5, BSA 0.1%, pH 7.4, 310 mOsm/l) (Li et al., 2004) and the cells were incubated under 95% air and 5% CO_2_ at 37°C for 2.5 h. In the second series of experiments, cardiac myocytes were subjected to SI by incubating the cells in hypoxic solution (in mM: NaCl 119, KCl 5.4, MgSO_4_ 1.3, NaH_2_PO_4_ 1.2, HEPES 5, MgCl_2_ 0.5, CaCl_2_ 0.9, Na-lactate 20, BSA 0.1%, 310 mOsm/l, pH = 6.4) (Li et al., 2004) and placing the plates in a humidified 37°C hypoxic chamber exposed to a constant flow of a mixture of 95% N_2_ and 5% CO_2_ for 4 h. The cells were then subjected to the following treatments during SI or normoxic protocol: vehicle control or MMP inhibitors at different doses calculated according to IC doses *in vitro*. Normoxic and SI treatments were followed by 2 h reoxygenation with growth medium with administration of the same dose of compounds as during normoxia or SI and superfusion with 95% air and 5% CO_2_ at 37°C (Figure 3).

#### Cell viability assay

Cell viability was assessed by a calcein and propidium iodine assay performed in each group after 2 h reoxygenation. Briefly, the growth medium was removed, the cells were then washed with PBS twice and afterwards were incubated with calcein (1 μM) for 30 min. Then the calcein solution was replaced with fresh PBS and the fluorescence intensity of each well was detected by a fluorescent plate reader (FluoStar Optima, BMG Labtech, Ortenberg, Germany). Fluorescent intensity was then measured in well scanning mode (scan matrix:10 × 10; scan diameter: 10 mm; bottom optic; no of flashes/scan point: 3; temp: 37°C; excitation wavelength: 490 nm; emission wavelength: 520 nm). Then the PBS was removed and the cells were incubated with PI (50 μM) and a digitonin (10^−4^ M) (Sigma-Aldrich; St. Louis, MO) for 7 min. Following that, the PI solution was replaced with fresh PBS and fluorescent intensity was detected using the same settings, excitation wavelength: 544 nm; emission wavelength: 610 nm). Background fluorescent intensity (Cells without staining) was subtracted from the calcein fluorescence intensity (reflecting live cell population) and divided by PI fluorescence intensity (reflecting total cell count) and the average intensity of each group was plotted. The cytoprotective effect of different compounds was compared to simulated ischemic control groups.

### Myocardial infarction in isolated rat heart

#### *Ex vivo* global ischemia/reperfusion injury

Our experiment conforms to the National Institutes of Health Guide for the Care and Use of Laboratory Animals (NIH Pub. No. 85-23, Revised 1996) and also to the EU directive guideline for the care and use of laboratory animals published by the European Union (2010/63/EU) and was approved by the local ethics committee of the University of Szeged. Eight to ten week-old male Wistar rats weighing 300–350 g (Toxicoop Ltd., Budapest, Hungary) were anesthetized intraperitoneally with 60 mg/kg pentobarbital sodium (Euthasol, Produlab Pharma, Raamsdonksveer, The Netherlands). After administration of 500 U/kg heparin through the femoral vein, the heart was isolated and perfused according to Langendorff with oxygenated Krebs-Henseleit buffer at 37°C as previously described (Turan et al., 2006). Briefly, hearts were subjected to 10 min aerobic perfusion for equilibration and stabilization of heart function and then by 30-min global ischemia followed by 120 min reperfusion. Global ischemia was induced by setting a stopcock (B/Braun, Melsungen, Germany) in closed position, and reperfusion was achieved by turning the stopcock in the original (perfusion) position. Heart rate and coronary flow were monitored throughout the perfusion protocol. All the test compounds, their vehicle (DMSO, <0.1% in Krebs-Henseleit solution) as well as the positive control PD166793 (Tocris Bioscience, Cat. No. 2520; Bristol, UK) were applied 20 min before the onset of global ischemia and maintained until the 30th min of reperfusion (Figure 3).

#### Determination of myocardial infarct size

At the end of the 2-h reperfusion, the right ventricle was removed, hearts were frozen, cut into six 1-mm-thick slices, and incubated in 1% triphenyl-tetrazolium chloride (Sigma-Aldrich; St. Louis, MO) at 37°C to delineate infarcted tissue. Slices were then fixed and quantified by planimetry using Infarctsize™ 2.5 software (Pharmahungary, Szeged, Hungary) (Fekete et al., 2013). Infarct size was expressed as a percentage of the left ventricle.

### Statistical analysis

Data were expressed as mean ± SEM. Cell viability were expressed as % of vehicle treated groups. Data were compared to vehicle using ANOVA followed by *post-hoc* tests, e.g., Tukey or Fisher LSD test.

## Results

### Focused library design and MTS screening

Since hydroxamic acids are reported as the primary zinc-binding motif, we intended to select such a library from the AMRI 200,000 member non-exclusive compound repository as a starting point of our drug discovery efforts. Since only a few compounds were available in the repository as hydroxamic acids and the conversion of acids to hydroxamic acids were not applicable to HT parallel synthesis, we decided first to select free acids from the AMRI's compound collection. This selection supported our initial hypothesis since acids are considered as weaker Zn^2+^ chelators than hydroxamic acids, which might be beneficial for achieving selectivity and in addition could be considered as a good indicator of the MMP-2 inhibitory activity. The substructure search resulted in 3600 acids, which were further focused to a small diverse subset by chemoinformatics methods including 259 compounds, where the compounds hold various motifs around a central core, reflecting the typical MMP inhibitor architecture described above (see Figure 2). The selected acids were screened in a fluorescent assay using a recombinant human MMP-2 catalytic fragment and a synthetic peptide substrate. Ilomastat (a non-selective MMP inhibitor) was used for the validation of the assay and in each subsequent experiment as a control compound. The selected compounds (259) were first tested using single point measurements at 10 μM concentration; 6 compounds showed > 70% inhibition, 7 compounds between 60–70%, and 12 compounds between 50–60%. The accumulated hit-rate was 10%. The primary acid hits (12) were attempted to convert to hydroxamic acids. Since two reactions failed 10 hydroxamic acids were prepared successfully for comparative MMP-2 screening. The hydroxamic acid pairs of the previously measured acids were then tested. Comparing the inhibitory activity of the acids and hydroxamic acids, we had an unexpected discovery. Five acids showed higher inhibition than the corresponding hydroxamic acids during catalytic fragment measurement, and among them 3 belonged to the same chemotype: thiazolyl-carboxylic acid (Table 1).

**Table 1:** Comparing the inhibitory activity of the acids and hydroxamic acids.

**Structure**	**Type**	**Code**	**IC_50_ on MMP-2 (μM)**	**Structure**	**Type**	**Code**	**IC_50_ on MMP-2 (μM)**
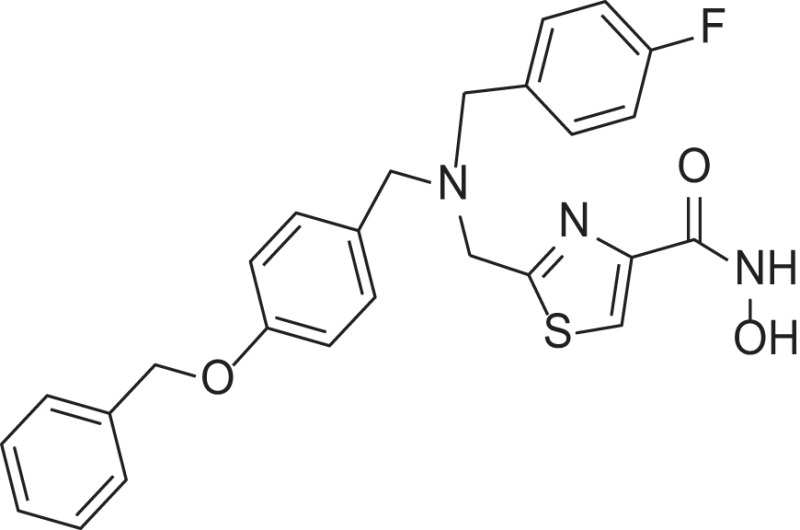	hydroxamic acid	AMRI-101H	12	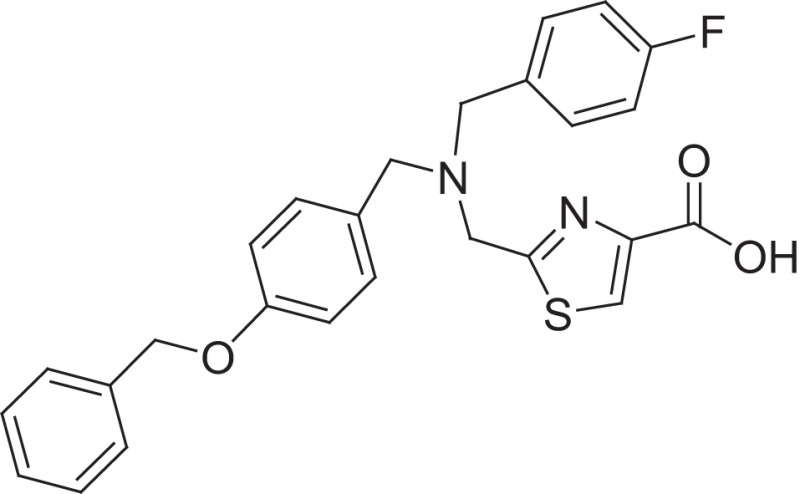	carboxylic acid	AMRI-101A/MMPI-1157	3.4
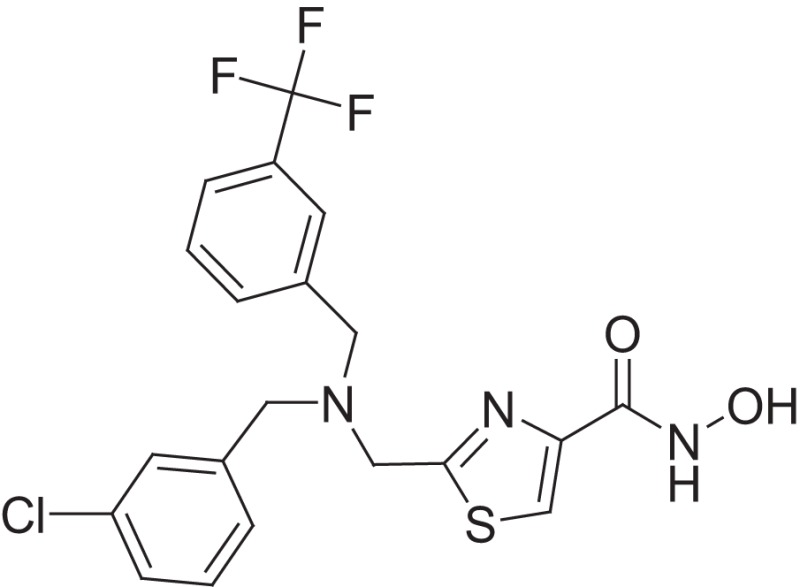	hydroxamic acid	AMRI-102H	11	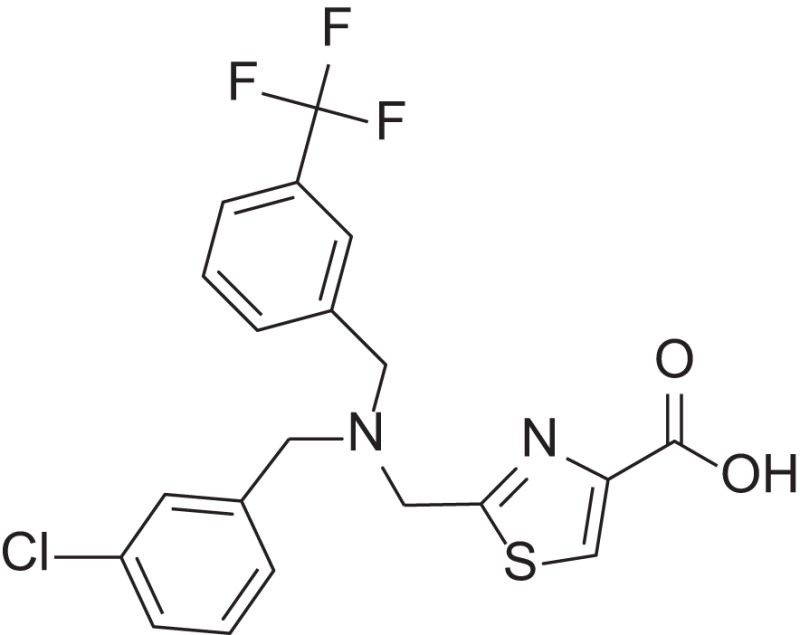	carboxylic acid	AMRI-102A	6.2
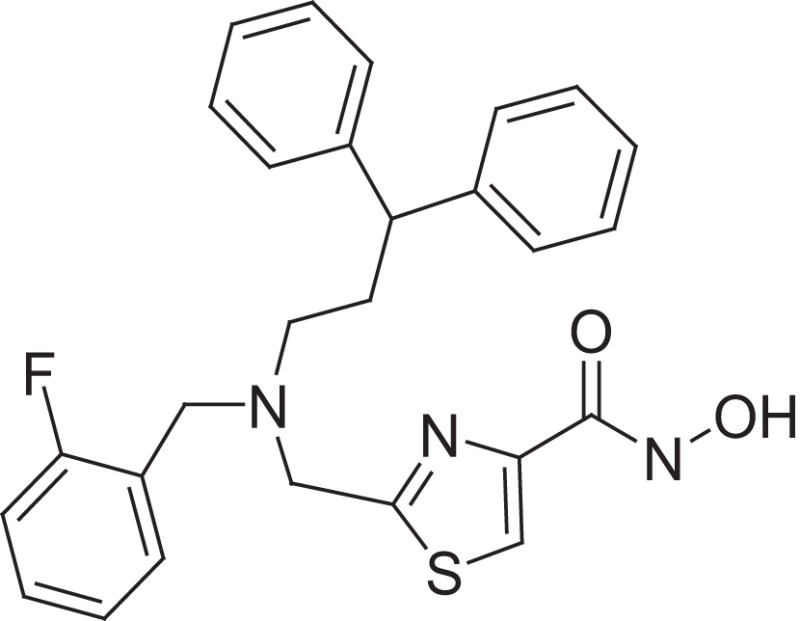	hydroxamic acid	AMRI-103H	17.6	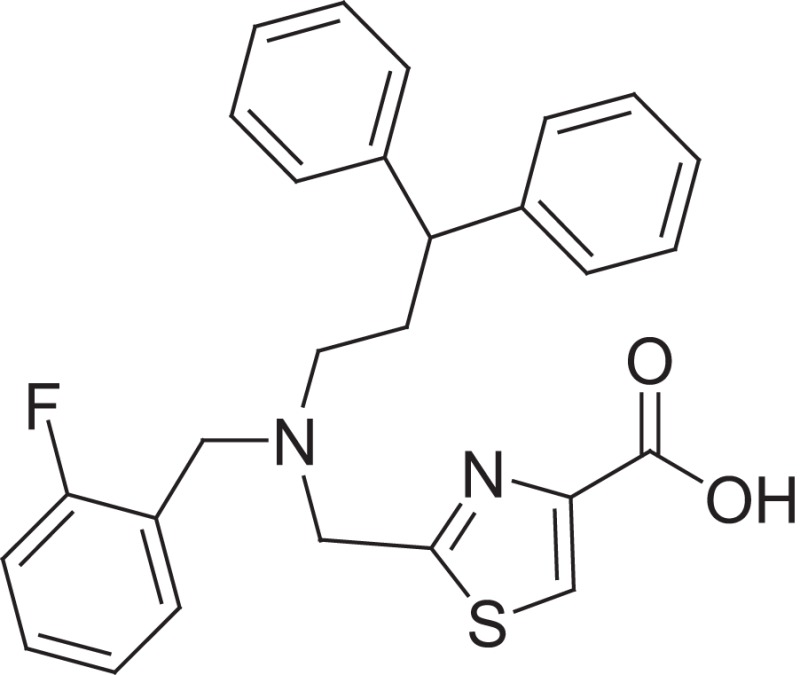	carboxylic acid	AMRI-103A	7.6

Furthermore, we found that the thiazole ring (MMPI-1157) to the isosteric imidazole (MMPI-1154) increased the selectivity to 1.5-fold over MMP-1 (Table 2) while the overall inhibitory profile was similar. The 3D similarity score was also high (3D-T = 0.85). The thiazole-imidazole replacement also made the compounds less lipophilic (cLogP was reduced from 3.3 to 2.9). Interestingly, 4- (or para)-fluoro-phenyl substitution in the shorter side chain (MMPI-1157, 1154, 1260, 1248) is favored over the 3- (or meta)-fluoro-phenyl substitution. It showed higher selectivity and MMP-2 inhibitory effect even if the 3D similarity scores were high. The 4- benzyl-phenyl ether or 4-pyridyl-phenyl ether side chain was also favored over the other groups in the longer side chain. On the other hand, if the benzene ring was replaced with pyridine in the shorter side chain, it reduced the MMP-9 inhibition significantly, thus MMP-2/9 selectivity was increased (MMP 9 inhibition: MMPI-1252, 1253 ≥ 500 μM). One compound (MMPI-1140) that lacks the heterocyclic ring but contains the corresponding side chains showed similar activity profile as the parent thiazole carboxylic acid, MMPI-1133, even though the 3D similarity alignment was relatively low (0.56). In summary, the entire screening cascade (Figure 2) including library design, selection, virtual screening, and *in vitro* biological screening resulted in a novel thiazole/imidazole carboxylic acid chemotypes, which could be suitable starting points for further structure-based optimization.

**Table 2:** Results of thiazole carboxylic acid (TCA) and imidazole carboxylic acids (ICA) and related analogs.

**Structure**	**Code**	**IC_50_ on MMP-1 (μM)**	**IC_50_ on MMP-2 (μM)**	**IC_50_ on MMP-9 (μM)**	**IC_50_ on MMP-13 (μM)**	**3D alignment to 1157**	**cLogP**
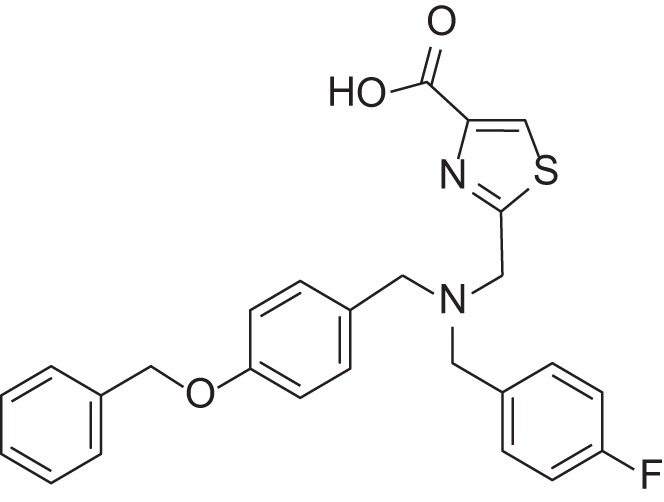	MMPI-1157 (TCA)	3.6	3.4	15	1.6	1.00	3.33
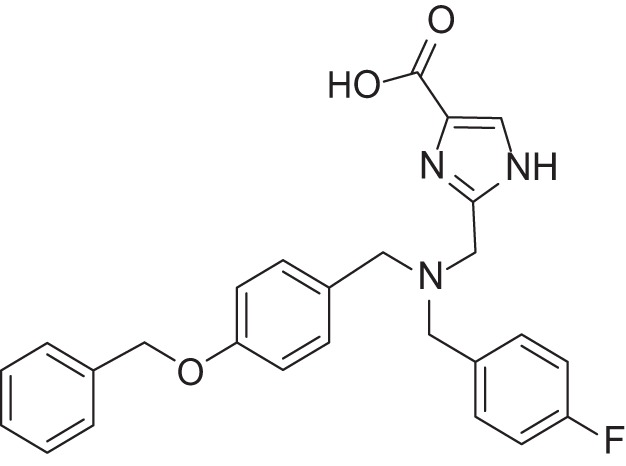	*MMPI-1154 (ICA)*	10	6.6	13	1.8	0.85	2.91
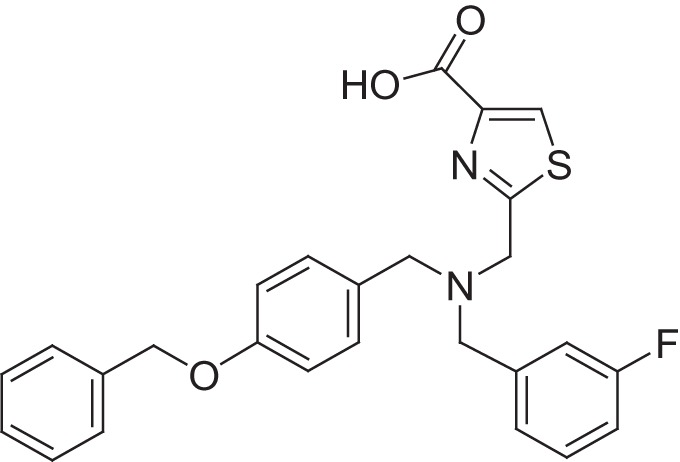	MMPI-1133 (TCA)	6.8	25	9,8	4.7	0.932	3.37
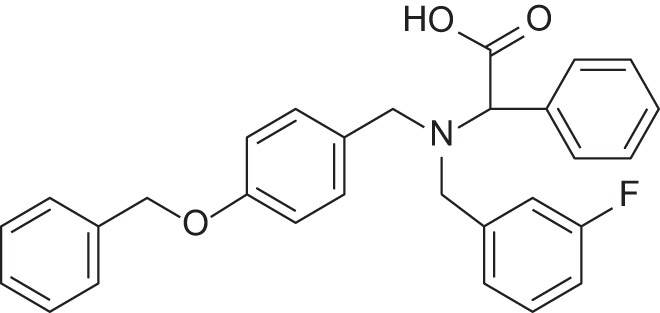	*MMPI-1140*	12	20	39	2.6	0.56	2.91
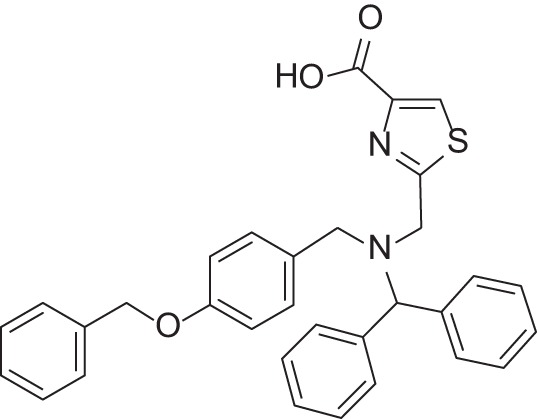	MMPI-1155 (TCA)	26	25	10	1.76	0.663	4.77
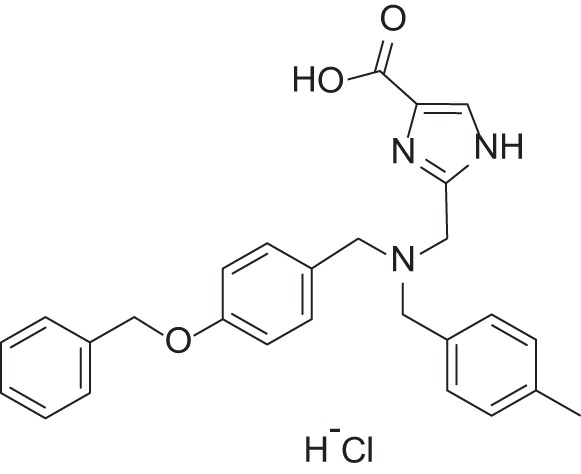	MMPI-1247 (ICA)	33	15	100	3.3	0.843	3.11
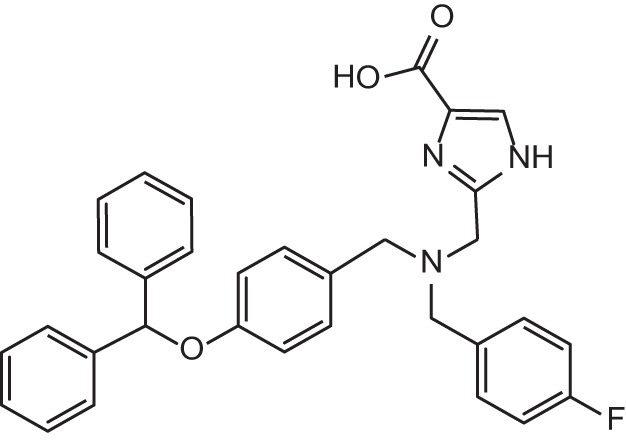	MMPI-1245 (ICA)	16	35	8	0.28	0.673	4.70
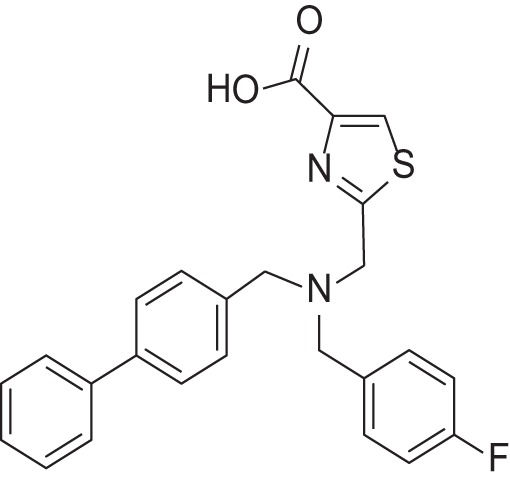	*MMPI-1254 (TCA)*	17	30	20	3	0.739	3.39
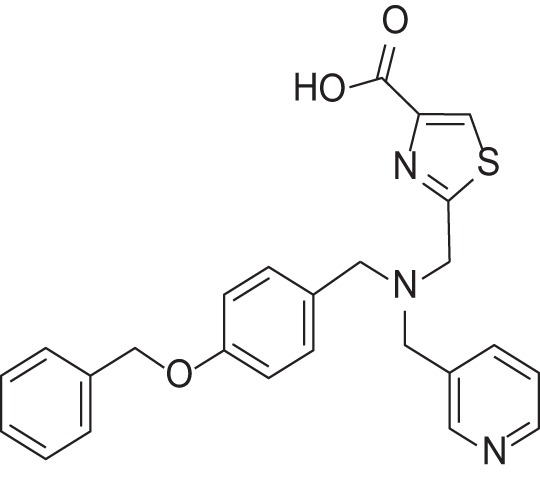	MMPI-1253 (TCA)	240	90	>500	8	0.927	2.22
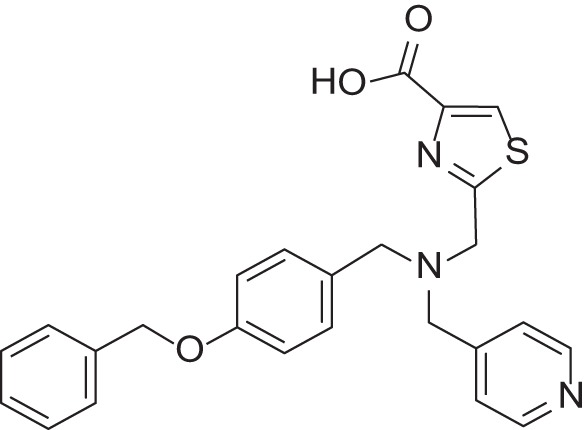	MMPI-1252 (TCA)	115	54	>500	1.5	0.948	2.20
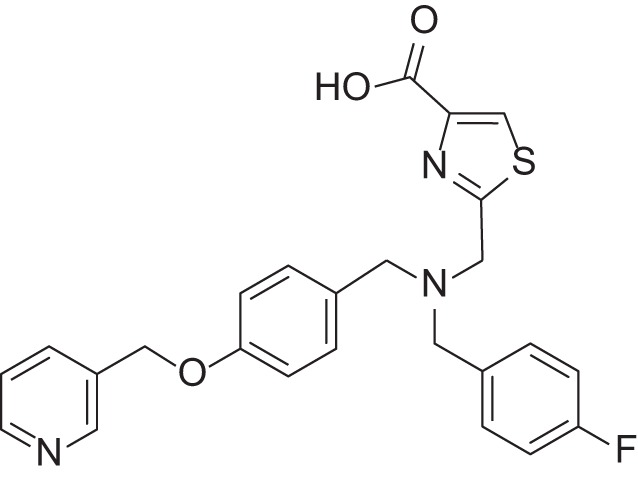	MMPI-1260 (TCA)	51	5.7	37	2.5	0.916	2.16
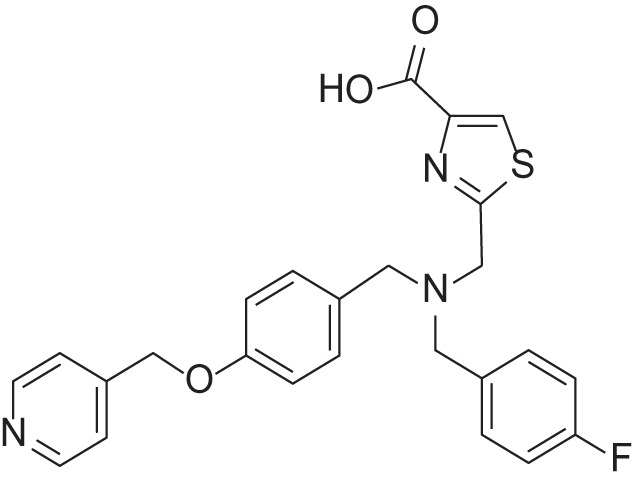	MMPI-1248 (TCA)	47	8	8.8	1.24	0.962	2.24

As a next step we started to explore the chemical space around this chemotype using 2D/3D structure-based *in silico* methods. First, a 568-membered focused library was *in silico* generated around the AMRI library hits including their bioisosters and some simplified analogs and then the library members were docked to the 3D model of MMP-2.

Virtual 3D docking of potential MMP inhibitors was executed using GOLD. The protein structure coordinates were obtained from Protein Data Bank using the highest available resolution (preferably co-crystallized with ligand). We used (MMP-2: 1QIB), (Dhanaraj et al., 1999). The region of interest used for GOLD docking was defined as all the protein residues within the 19 Å radius sphere with the midpoint of the Zinc ion in the catalytic center. GOLD default parameters were used, which were set to 200,000. The complexes were submitted to 20 genetic algorithm runs using the GOLDScore fitness function.

As a result, 45 compounds were considered as virtual hits (docking score > 70) and proposed for chemical synthesis. The synthesizable compound set was completed with several close analogs by rational design. For instance, in order to increase the solubility of the compounds, the benzene ring was successively replaced with pyridine (see MMPI-1252, 1253, 1248, and 1260). Altogether 30 compounds were successfully synthesized for screening combining the *in silico* hits and the additional designed compounds.

The compounds were measured for MMP-1, 2, 9, 13 to determine their inhibitory profile. Efficiency Index amplifies the two major required effects, selectivity against MMP-1 and the inhibitory activity.

Table 2 shows the IC_50_ values of the hit compounds (hit criteria: 100% MMP-2 inhibition at 100 microM). The Gold docking scores are shown for those hits that are coming from virtual screening.

In addition, 3D flexible alignment studies were performed between the novel hit compounds and the initial AMRI library best hit (AMRI-101A/MMPI-1157) compounds. The measure of the alignment was characterized by 3D similarity scores (3D Tanimoto coefficient, ChemAxon Screen3D software). It was postulated that high 3D similarity score could reveal similar conformation and binding mode which could result in similar bioactivities. Finally, cLogP was calculated for each compound. The lower values showing less lipophilicity which is expected to accelerate the passage through the cell membrane leading to higher bioavailability.

**Figure 4:**
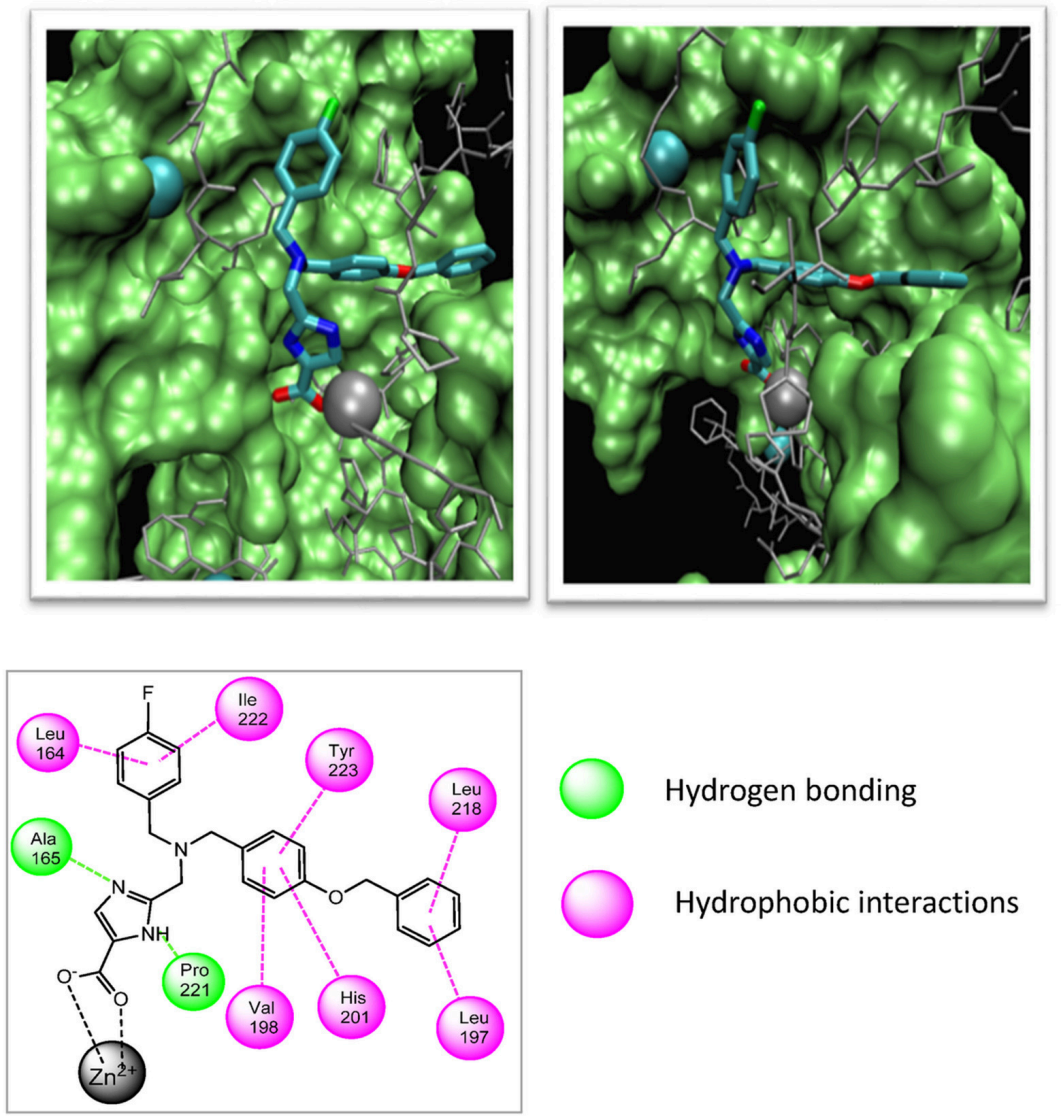
Two different views of the 3D structure of MMP-1154 docked to the active site of MMP-2 together with the major binding interactions.

MMPI-1154 was investigated more deeply in 3D docking studies. Figure 4 shows the interaction of the compound to the active site of MMP-2. In MMPI-1154 (Containing an imidazole-carboxylic acid moiety), the acid residue had a chelating interaction to the Zn^2+^ with the contribution of one of the N-hetero atoms of the heterocyclic ring. This relatively weak Zn^2+^ chelation dynamically and statistically gives an allosteric binding feature of this inhibitor.

### The effect of MMP inhibitors on cardiac MMP-2 activity measured by zymography

To confirm the MTS screen results, we tested the potential MMP inhibitor molecules on MMP-2 enzyme isolated from rat heart *in vitro*. Therefore, we applied the MMPIs at 1 and 100 μM final concentration in the enzyme's development buffer (Table 3).

**Table 3:** Screening of molecules on cardiac MMP-2 with gelatin zymography.

**Code**	**Inhibition (%)±SEM at 100 μM final MMP inhibitor concentration**
MMPI-1133	11.14 ± 1.58
MMPI-1140	81.08 ± 3.88
MMPI-1154	100
MMPI-1155	46.95 ± 19.06
MMPI-1157	100
MMPI-1245	100
MMPI-1247	100
MMPI-1248	100
MMPI-1252	100
MMPI-1253	100
MMPI-1254	100
MMPI-1260	100

### Cardio-cytoprotection by MMPIs in cell culture model of I/R injury

Some doses of MMPIs affected cell viability significantly in normoxic conditions (Supplementary Figure 4). Since the vehicle for MMPIs was DMSO, aerobic cardiac myocytes were treated with 0.1% (v/v%) DMSO and their viability was also assessed. Vehicle treatment did not affect cell viability in comparison to non-treated cardiomyocytes (Supplementary Figure 5).

Hypoxia is one of the numerous influences on cardiac matrix remodeling, via ECM turnover and induction of MMPs. In addition, I/R injury is also a critical modulator of MMP expression through alternative mechanisms (Jun et al., 2011).

**Figure 5:**
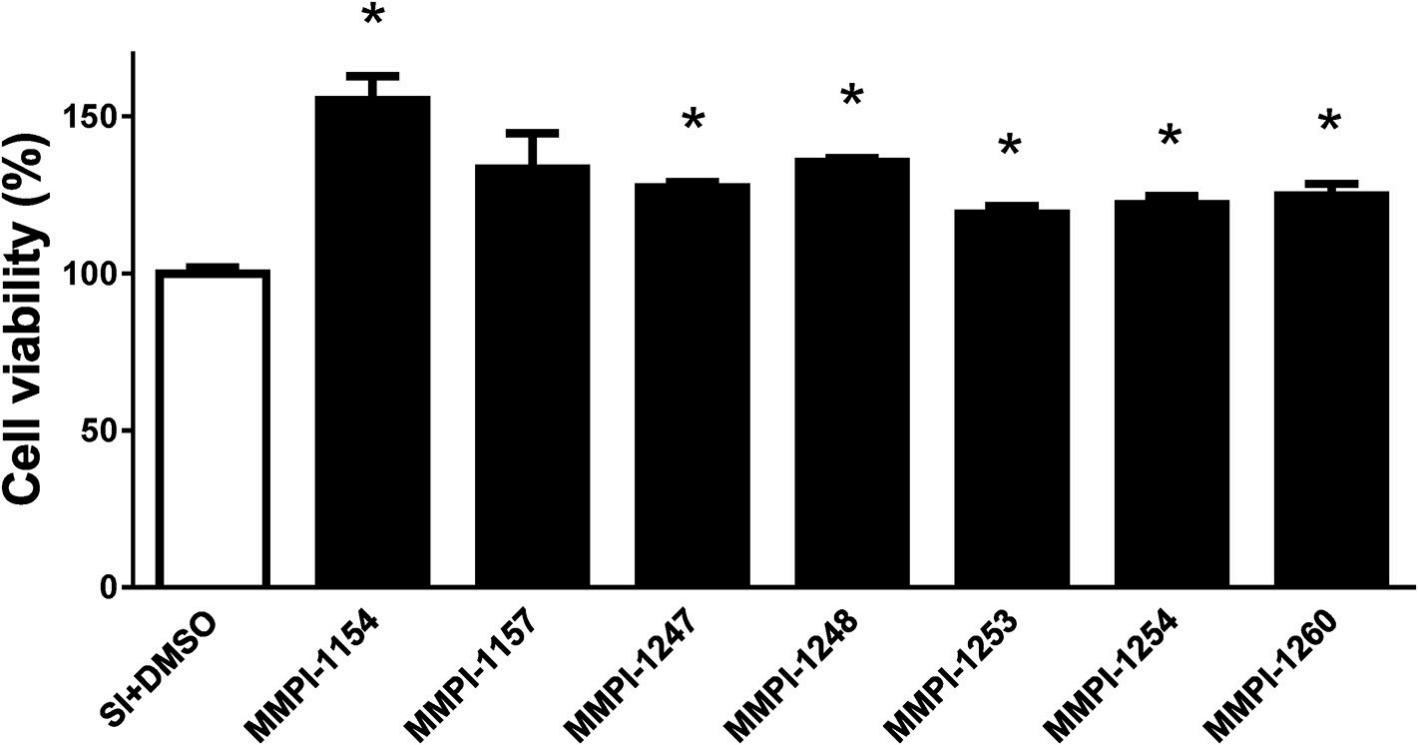
Cardioprotective effects of MMPI lead candidates on neonatal rat cardiac myocytes subjected to simulated I/R. Cell viability was measured after 4 h simulated ischemia followed by 2 h of simulated reperfusion. Data are expressed in the ratio of vehicle (DMSO) control in percentage. Positive data (more than 100%) shows higher viability compared to the control. ^*^p < 0.05 vs. Vehicle, *n* = 5–6 (One-way ANOVA followed by Dunnett *post-hoc* test). The most effective doses of the series of experiments are presented in the case of all compounds (for more detailed results see for Supplementary materials, Figure 4).

The 4-h hypoxic exposure and 2-h reoxygenation caused a marked cell death (Supplementary Figure 5), which was attenuated by MMPI treatment. To investigate whether MMPIs treatment influences cardiac myocite survival after simulated I/R, we selected 6 MMPIs that were available at that time and, which showed significant MMP inhibitory effect during pre-screening. We tested those compounds in cultured neonatal cardiac myocytes subjected to simulated I/R studies. Ilomastat served as positive control (Supplementary Figure 6). The tested compounds showed significant cytoprotection, between 17 and 47% (Figure 5). The supplementary figures show all inhibitor testing data (Supplementary Figure 4).

### Cardioprotection by MMPI-1154 in isolated rat heart model of I/R injury

**Figure 6:**
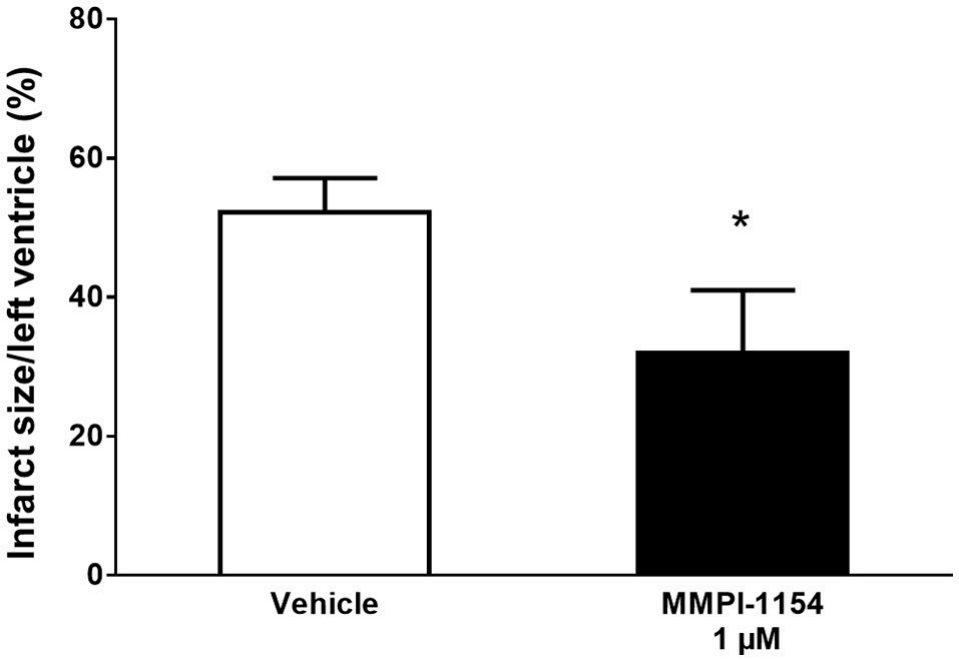
MMPI-1154 is cardioprotective. The effect of MMPI-1154 on myocardial infarct size in isolated rat hearts subjected to 30 min global ischemia followed by 120 min reperfusion. ^*^p < 0.05 vs. Vehicle, *n* = 6–8 (One-way ANOVA followed by Fisher LSD *post-hoc* test).

Finally, based on the results of cell culture experiments, we selected the most potent cardioprotective compound, MMPI-1154 for testing in an isolated rat heart model of AMI. MMPI-1154 reduced myocardial infarct size significanly at 1 μM as compared to the vehicle-treated group (Figure 6).

## Discussion

In our study, we have successfully demonstrated the development of a novel, selective MMP-2 inhibitor for cardioprotection from an *in silico* compound library selection, through to the testing of the most promising compound against acute myocardial infarction, in an isolated rat heart model. We've found that the MMP-inhibiting effects of imidazole and thiazole carboxylic acid-based compounds are superior to the conventional hydroxamic acid type derivatives of the same molecules. We have thus shown for the first time in the literature that the acute application of MMPI-1154 (An imidazole carboxylic acid-based compound) has a protective effect for the heart against acute myocardial infarction. We achieved *ex vivo* cardioprotection via a moderate MMP-2 inhibition, since MMPI-1154 was applied at around the concentration of its IC_20_ value.

### MMP inhibitor development strategy

Currently, ~500 papers investigating the role of MMP inhibition in myocardial ischemia are available from the last 2 decades in PubMed database. There are several papers that describe the non-zinc binding, allosteric (e.g., π-π stacking) interactions of MMP-2 with selected inhibitors (Di Pizio et al., 2013; Agamennone et al., 2016; Ammazzalorso et al., 2016; Adhikari et al., 2018). Most of these papers employ MMP-2 as a potential biomarker for ischemic heart diseases or as a therapeutic target to evoke cardioprotection. However, early clinical trials targeting MMP-2 for improving cardiovascular outcomes after acute myocardial infarction have failed (e.g., PREMIER study, Hudson et al., 2006). The likely reason for failure was the lesser selectivity of the applied MMP inhibitors as well as the chronic and relatively high-dose administration regimen. Therefore, in our present study, we aimed to develop novel MMP-2 inhibitor lead candidates, which possess high selectivity and lead only to a moderate MMP-2 inhibition in accordance to our previous findings (Giricz et al., 2006; Bencsik et al., 2014).

### Novel structural findings regarding MMP-2 inhibitor development

Several hydroxamic acid compounds are known as non-selective MMP inhibitors. Therefore, we started our inhibitor development with selecting hydroxamic acid compounds from the AMRI library. We also selected their carboxylic acid derivatives. We identified thiazole and imidazole substituted carboxylic acid molecules, in which MMP-2 inhibitory effect was superior to the corresponding hydroxamic acid derivatives. Furthermore, we found that changing the thiazole ring (MMPI-1157) to the isosteric imidazole (MMPI-1154) increased the selectivity over MMP-1, although the overall inhibitory profile and the structure were similar. This feature was an advantageous factor during molecular designing process since MMP-1 inhibition was responsible for the development of musculoskeletal syndrome, the most severe adverse effect of early MMP inhibitors.

The relatively weak Zn^2+^ chelation derived from the imidazole-carboxylic acid moiety interacting to the Zn^2+^ dynamically and statistically gave an allosteric binding feature for MMPI-1154. It is also assumed that the additional electron donating heteroatom being in close proximity to the acid moiety (thiazole/imidazole ring) would also contribute to the chelation of the Zn^2+^ ion. The bulky side chain is deep inside in the S1' pocket as expected, although some rotational movements would be permitted around the central tertiary N atom. This option would allow different binding modes and activity profiles as well.

Most importantly, the pyridine moiety instead of the phenyl ring at the end of the S1' pocket occupying longer side chain of the molecules increased the selectivity of the inhibition for MMP-2 against MMP-1 (MMPI-1260, 1248). This is most likely due to the increased polarity of the tail group (such as pyridine), which is exposed to the aqueous environment at the end of the S1' pocket. Similar compounds are described in Duan et al. (2007), where non-zinc chelating MMP-2 inhibitors with a similar bulky side chain were reported. This finding supported our hypothesis that weak or negligible Zn^2+^ chelation with bulky and partially polar side chains lead to selective and active MMP-2 inhibitors. The phenyl-pyridine exchange is also beneficial to the cell penetration since the calculated octanol-water partition (cLogP) decreased in one order of magnitude. Although this change did not cause significant conformational changes, the 3D similarities were high between these compounds and the initial hit (MMPI-1157).

In conclusion, the biological data and the docking studies together with the 3D alignment modeling confirmed that these chemotypes represent a novel promising class of MMP-2 inhibitors. The bulky groups together with a weaker Zn^2+^-chelating carboxylic acid residue allowed us to achieve low micromolar MMP-2 inhibition, often together with an apparent selectivity against MMP-1. Finally, all the hit compounds meet the drug-likeness criteria (Lipinski Rule of 5.), which predicts high developability prognosis.

### Screening cascade

After the chemical optimization of the novel MMP inhibitor lead candidates, we determined their IC_50_ values by using gelatin zymography. During zymographic analysis, we used full-length, active MMP-2 enzymes isolated from healthy young adult rat hearts. Subsequently, the cardio-cytoprotective effects of the selected candidates having the lowest IC_50_ values to MMP-2 were tested in cultured neonatal cardiac myocytes subjected to simulated I/R injury. Cardiac myocyte cell culture assay allowed a relatively high throughput biological efficacy testing (Gorbe et al., 2010) of the selected lead candidates in several dose ranges at different levels of inhibition of MMP-2 activity. Our cell culture test system revealed several biologically efficacious doses beyond the IC_50_ values of the selected lead candidates (see data Supplementary Figure 1 for details).

### Cardio-cytoprotection by MMPI-1154

Based on the results of the abovementioned cell culture experiments, we selected MMPI-1154 (The lead candidat) which showed the highest increase in cell viability during simulated I/R experiments. We then used it for cardioprotection in an *ex vivo* rat heart model of acute myocardial infarction. To approximate the moderate 20% inhibition of MMP-2 activity by MMPI-1154 (based on our previous findings, Giricz et al., 2006; Bencsik et al., 2014), in the *ex vivo* model of AMI, we used the 1 μM concentration (IC_20_ value) instead of the most effective 2.5 μM (IC_50_ value) concentration seen during cell culture experiments. Although MMPI-1154 is not highly selective to MMP-2, it seems to be one of the most efficient MMP-2 inhibitors as shown in Table 2 (efficiency index). In the present study, the *in silico* and subsequent *in vitro* chemical efficiency has been confirmed in the isolated heart experiments since MMPI-1154 in 1 μM showed a significant cardioprotection effect by decreasing myocardial infarct size during acute global ischemia/reperfusion injury. Further research in *in vivo* models of AMI can shed light on its cardioprotective properties as well as on its safety derived from the optimal selectivity toward different MMP isoforms.

## Conclusions

This is the first demonstration that imidazole and thiazole carboxylic acid-based compounds are more efficacious than their hydroxamic acid derivatives in MMP-2 inhibition. MMPI-1154 is a promising novel cardio-cytoprotective imidazole-carboxylic acid MMP-2 inhibitor lead candidate for the treatment of acute myocardial infarction.

## Author contributions

PB: Study management and paper writing; KK: Gelatine zymography and *ex vivo* experiments; AG and ZV: Cell culture experiments; ÉK: Paper writing, statistics, data, and figure management; JP and RG: Cell culture and *ex vivo* experiments; LK: *In silico* drug design, synthesis of analogs; LW: MMP inhibitor design, medical chemistry advising; FT: Synthesis of analogs; IH, GF, SC, and LB: Molecular docking and high troughput screening; TC and CC: Supervising zymography and *ex vivo* experiments; GD: Supervising *in silico* and HT screening, granting, paper writing; PF: Supervising whole project, paper writing, and granting.

### Conflict of interest statement

PB and AG are employed by and PF is the CEO of Pharmahungary 2000 Ltd; TC and CC were employed by Pharmahungary 2000 Ltd; FT was employed by and LK was the CEO of Infarmatik Ltd; LW is the CEO of OntoChem GmbH; IH, GF, and GD are employed by and SC is the CEO of Targetex Ltd; PF, TC, CC, KK, LK, FT, SC, IH, GD, and AG are the inventors of the patent WO_2012/080762_A1 and related national patents now assigned by Pharmahungary and TargetEx. The other authors declare that the research was conducted in the absence of any commercial or financial relationships that could be construed as a potential conflict of interest.
