# Supplementary material for: Development of Matrix Metalloproteinase-2 Inhibitors for Cardioprotection
Source: Front Pharmacol. 2018 Apr 5;9:296. doi: 10.3389/fphar.2018.00296 (PMC5896266; doi:10.3389/fphar.2018.00296)
Supplement: Supplementary file 1 [file Image1.pdf]

Supplementary materials

Supplementary Figure 1:

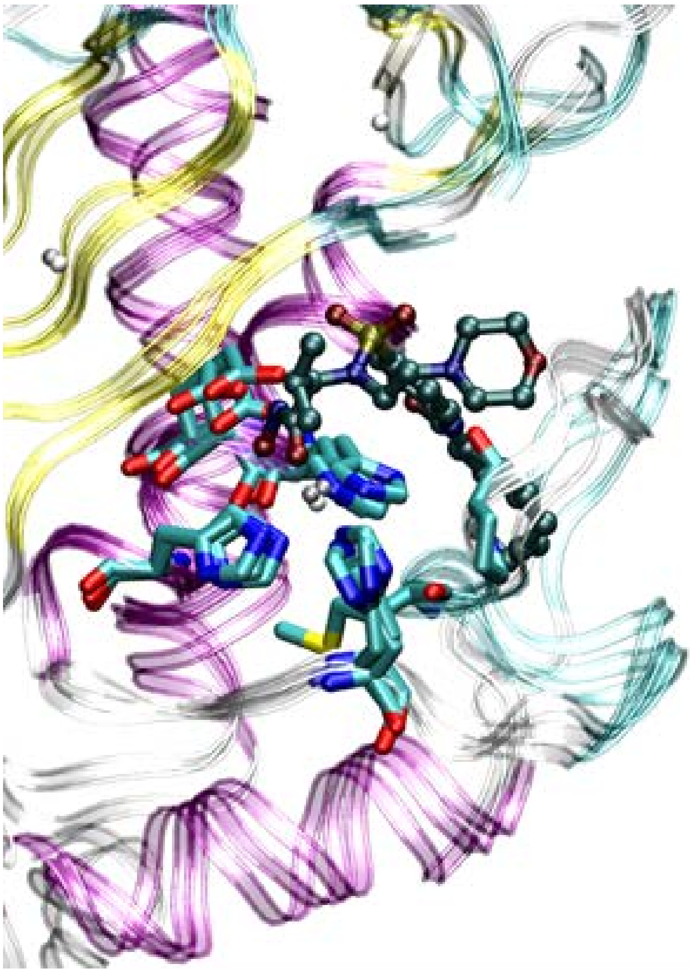

Superposition of the 3 MMP-2 model structures (PDB: 1EAK, 1CK7, 1HOV) in ribbon presentation. The peptide side-chains are shown (in colored according to the atom types) around the Zn-ions (small gray balls) In the 1HOV structure the SC-74020 inhibitor is shown. (originated from the NMR structure of the 1HOV - SC-74020 complex).
