# Supplementary material for: Development of Matrix Metalloproteinase-2 Inhibitors for Cardioprotection
Source: Front Pharmacol. 2018 Apr 5;9:296. doi: 10.3389/fphar.2018.00296 (PMC5896266; doi:10.3389/fphar.2018.00296)
Supplement: Supplementary file 2 [file Image2.pdf]

Bencsik et al., Cardioprotection by MMP-2 inhibition, *Front Pharmacol*, 2018

Supplementary Figure 2:

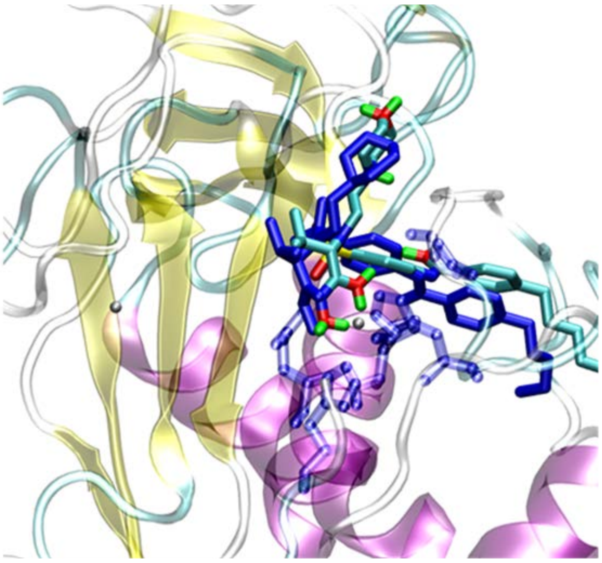

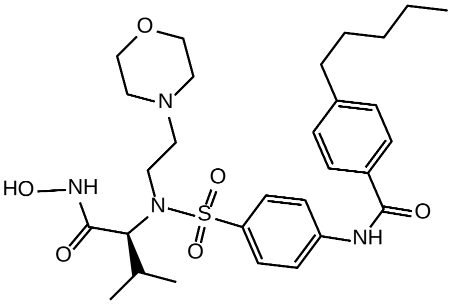

SC-74020, MMP-2 inhibition: 0.4 nM

SC-74020 docked into the 1EAK 3D structure (the blue structure is the conformation of SC-74020 based on the NMR structure of the 1HOV – SC-74020 complex for comparison). Gold docking (fitness) scores for ABT-518 was 95.55 and for PD-166793, 83.51. Gold docking score characterizes the level of fitness of the docking and correlates with the binding strength (and activity).
