# Supplementary material for: Development of Matrix Metalloproteinase-2 Inhibitors for Cardioprotection
Source: Front Pharmacol. 2018 Apr 5;9:296. doi: 10.3389/fphar.2018.00296 (PMC5896266; doi:10.3389/fphar.2018.00296)
Supplement: Supplementary file 3 [file Image3.pdf]

**Supplementary Figure 3:**

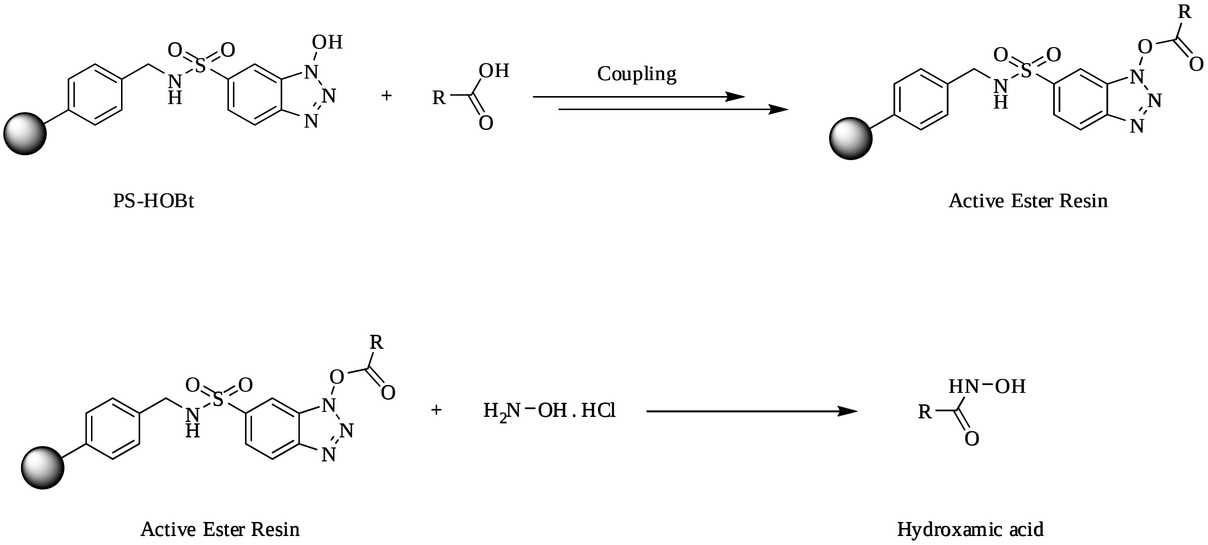


Schematic figure representing the synthesis of hydroxamic acids (see the text below for details)

Synthesis of hydroxamic acids

We developed a parallel synthesis procedure using resin-capture activation of the acids. The activated ester resin was converted to hydroxamic acids as shown below. After primary biological screening the acid „hits” (>60% inhibition at 10 mmol concentration) were chosen for hydroxamic acid generation.

General procedure for parallel synthesis of hydroxamic acids

The carboxylic acid was dissolved in abs. DMF and bromo-tris-pyrrolidino phosphoniumhexafluorophosphate (PyBrOP, 1 eq.), diisopropylethylamine (2 eq.) and PS-HOBt (polystyrene hydroxybenzotriazole) resin (0.6 eq., 1.35 mmol/g, pre-treated with abs. DMF) was added to the solution. The reaction mixture was shaken at r.t. for 3-4 hrs and the activated ester containing resin was filtered and washed three times with abs. DMF. This activation step was repeated under the same condition. After the second step the active ester resin was washed five times with abs. DMF. Hydroxylamine hydrochloride (1 eq. respect to the active resin) was dissolved in abs. DMF and diisopropilamine (1 eq.) was added together to the mixture containing the polymer-bound activated ester and the reaction mixture was shaken at r.t. overnight. After the reaction completed, the resin was filtered and washed with

abs. DMF three times. The filtrate was concentrated under reduced pressure and the residue was dissolved in DCM. The solution was extracted with 5% aq. $K_2CO_3$ solution and water. The organic layer was separated and evaporated to dryness. The crude product was dried and analyzed with LCMS and purified by prepHPLC if the purity did not reach 85 %.

The isolated yields were between 10- 76%, while the purity was higher than 85%. The compounds were obtained in 6-30 mg amounts. The success rate was 76%.
