# Supplementary material for: Development of Matrix Metalloproteinase-2 Inhibitors for Cardioprotection
Source: Front Pharmacol. 2018 Apr 5;9:296. doi: 10.3389/fphar.2018.00296 (PMC5896266; doi:10.3389/fphar.2018.00296)
Supplement: Supplementary file 4 [file Image4.pdf]

Bencsik et al., Cardioprotection by MMP-2 inhibition, *Front Pharmacol*, 2018

Supplementary Figure 4:

A#1

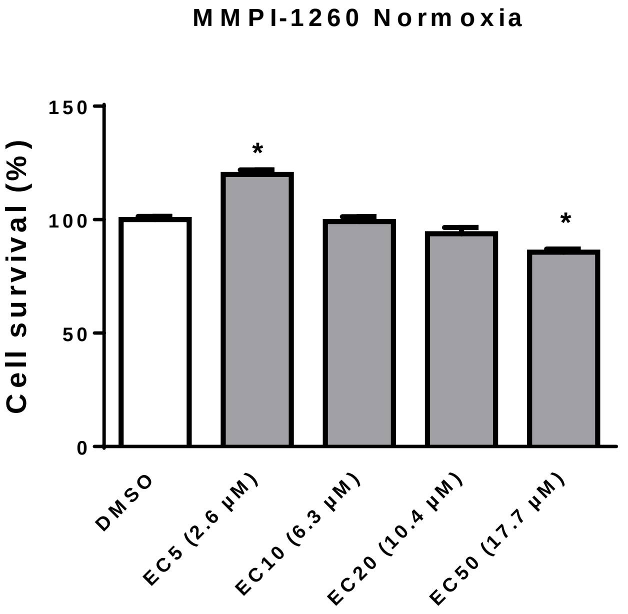

A#2

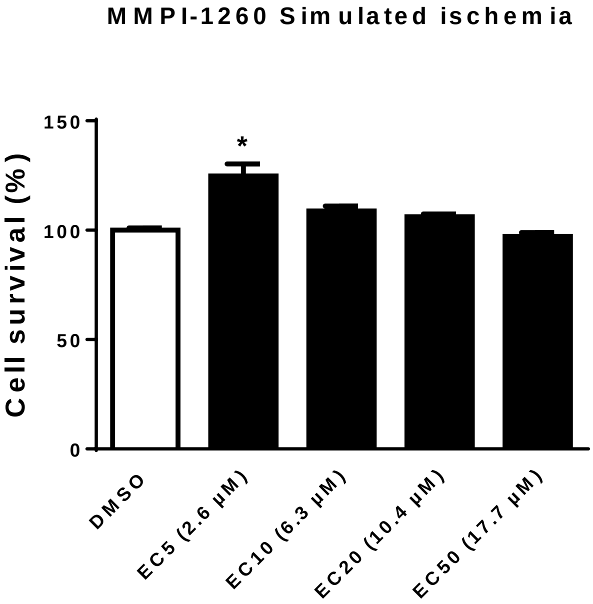

B#1

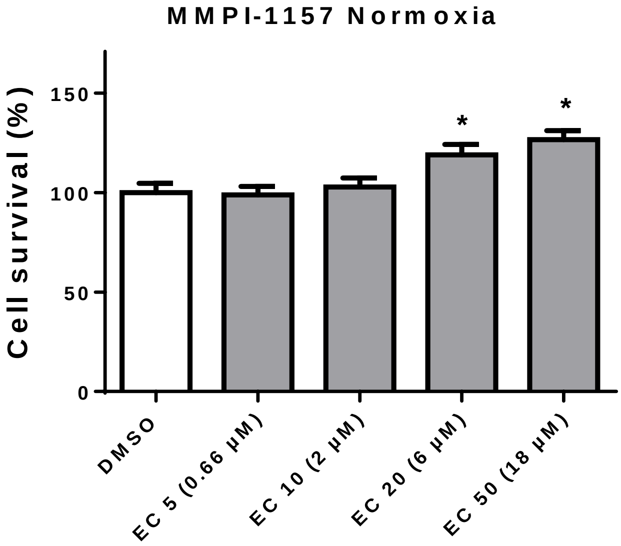

B#2

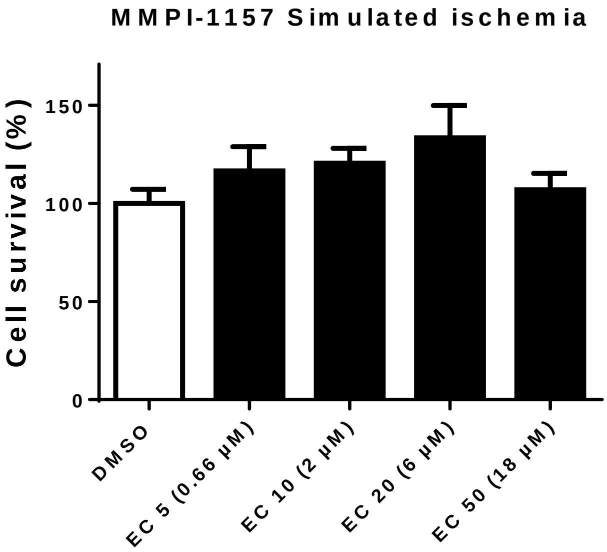

C#1

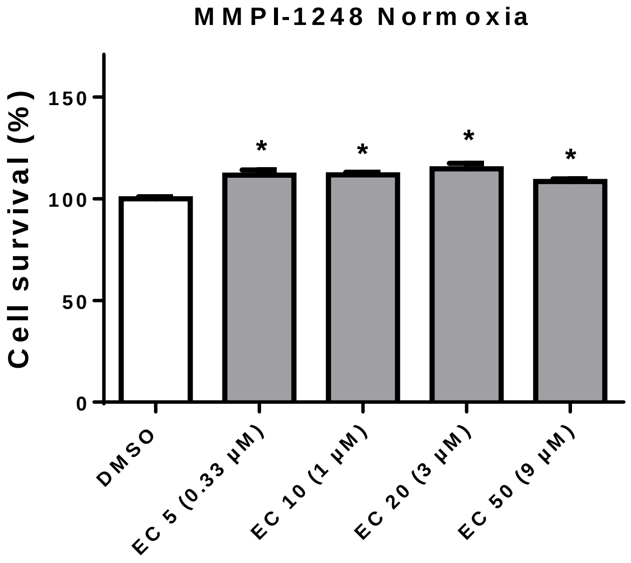

C#2

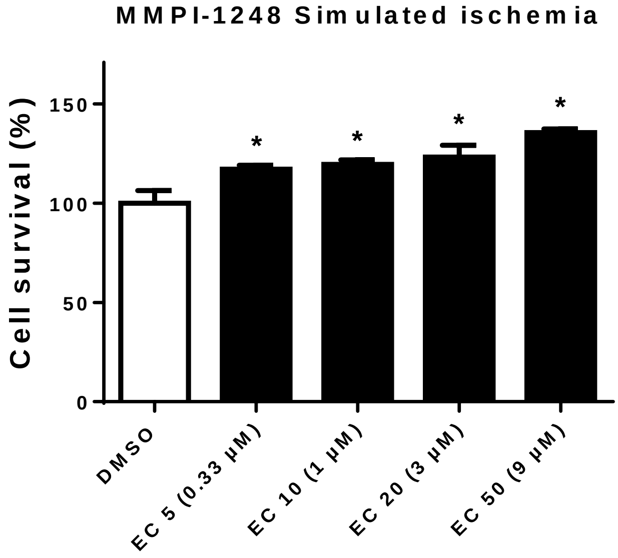

D#1

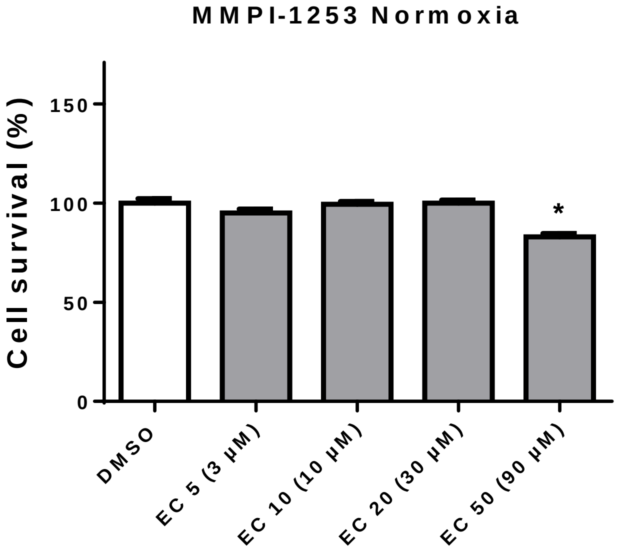

D#2

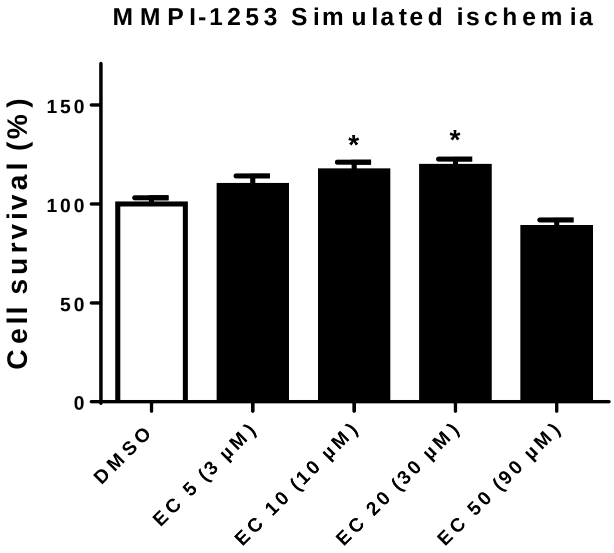

E#1

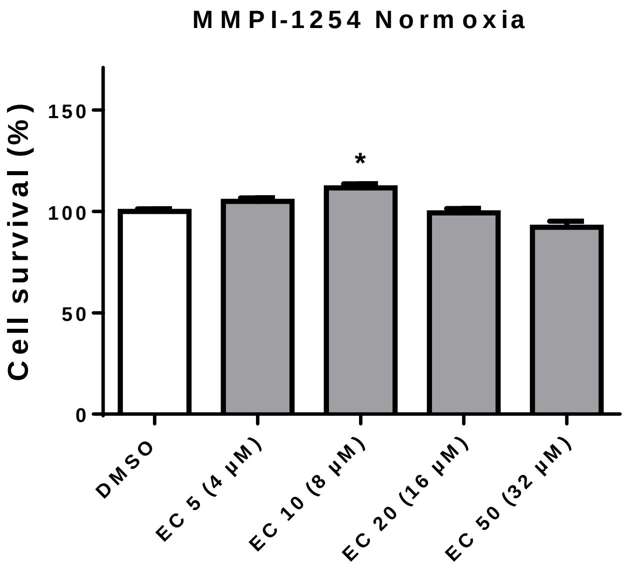

E#2

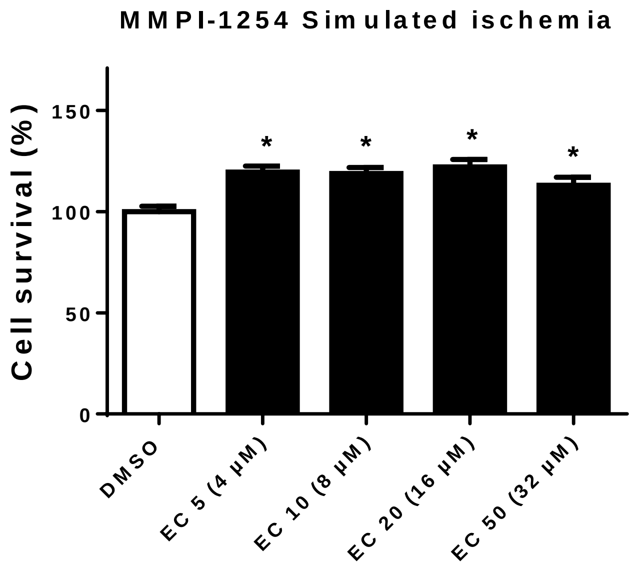

F#

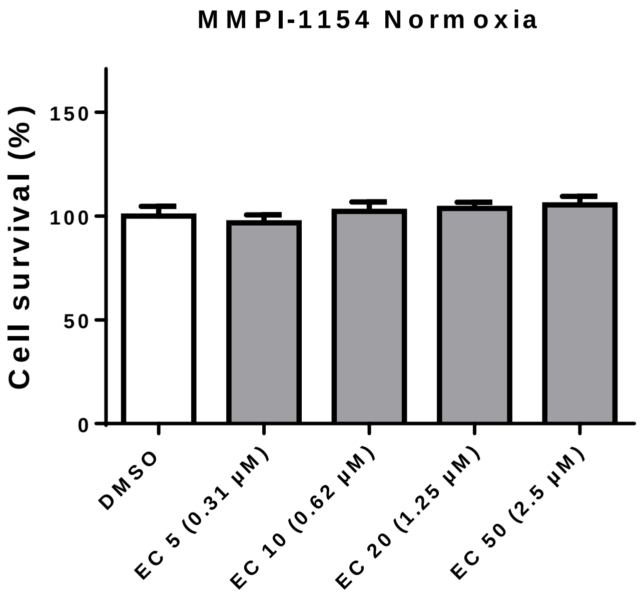

F#2

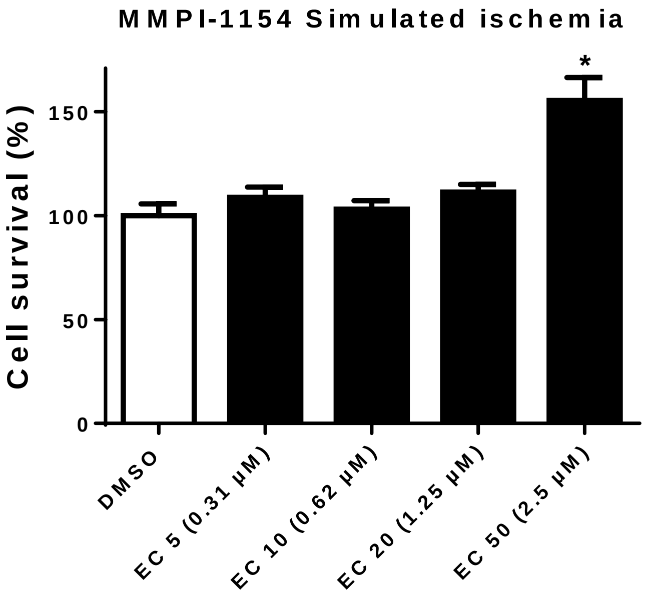

G#1

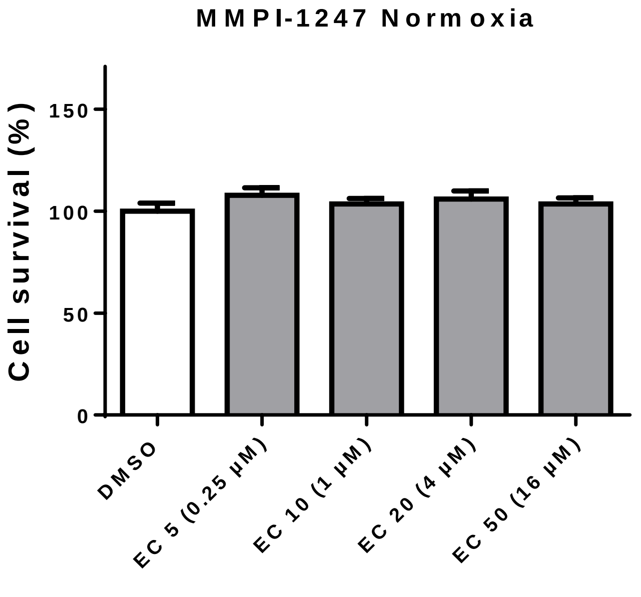

G#2

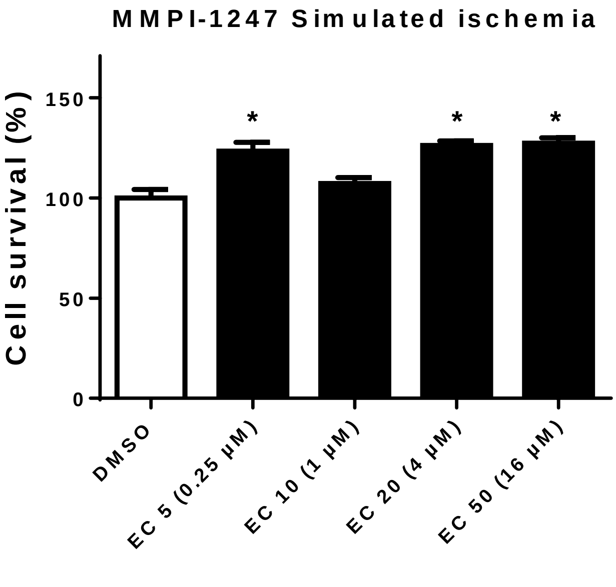

MMP inhibitor screen on neonatal rat cardiac myocytes.

Effect of each MMPIs (at EC 5, 10, 20, 50 concentration) on cardiac myocyte cell viability after 4 hours normoxia followed by 2 hours of simulated reperfusion (subpanels #1) and 4 hours simulated ischemia followed by 2 hours of simulated reperfusion (subpanels #2). Data are expressed in the ratio of vehicle (DMSO) control in percent. Positive data (more than 100%) shows higher viability compared to the control. Panel A: MMPI-1260; Panel B: MMPI-1157; Panel C: MMPI-1248; Panel D: MMPI-1253; Panel E: MMPI-1254; Panel F: MMPI-1154; Panel G: MMPI-1247. $^{*}p<0.05$ vs. Vehicle (DMSO), n=5-6 (One-way ANOVA followed by Dunnett post hoc test).
