# Supplementary material for: Development of Matrix Metalloproteinase-2 Inhibitors for Cardioprotection
Source: Front Pharmacol. 2018 Apr 5;9:296. doi: 10.3389/fphar.2018.00296 (PMC5896266; doi:10.3389/fphar.2018.00296)
Supplement: Supplementary file 5 [file Image5.pdf]

Bencsik et al., Cardioprotection by MMP-2 inhibition, *Front Pharmacol*, 2018

Supplementary Figure 5:

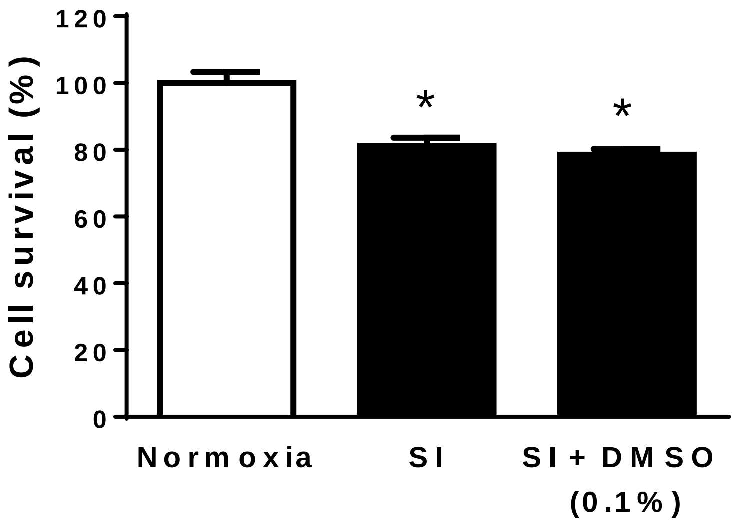

Effect of simulated ischemia/reperfusion injury on cell survival and effect of vehicle

Effect of 4 hours simulated ischemia followed by 2 hours of simulated reperfusion on cardiac myocyte cell viability in comparison to 4 hours normoxia followed by 2 hours of simulated reperfusion. Effect of 0,1% DMSO (vehicle) on cell viability. Data are expressed in the ratio of normoxic group in percent. *$p<0.05$, (One-way ANOVA followed by Dunnett post hoc test).
