# Supplementary material for: Development of Matrix Metalloproteinase-2 Inhibitors for Cardioprotection
Source: Front Pharmacol. 2018 Apr 5;9:296. doi: 10.3389/fphar.2018.00296 (PMC5896266; doi:10.3389/fphar.2018.00296)
Supplement: Supplementary file 6 [file Image6.pdf]

Bencsik et al., Cardioprotection by MMP-2 inhibition, *Front Pharmacol*, 2018

Supplementary Figure 6:

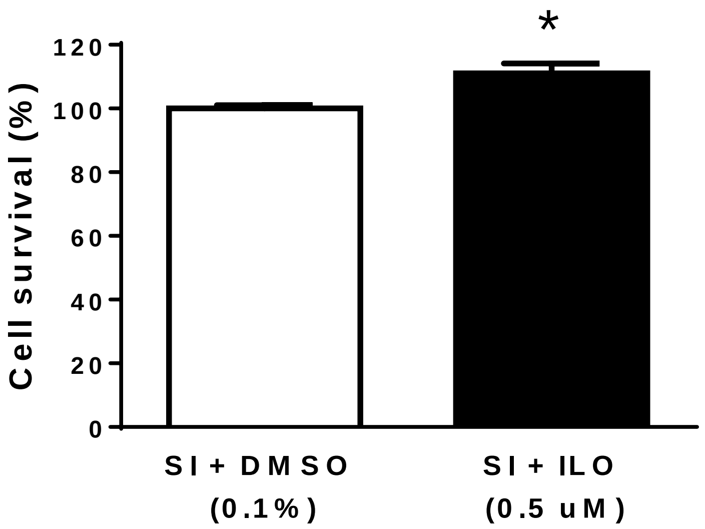

Effect of positive control ilomastat on cell survival

Effect of MMPIs Ilomastate (at 0,5 uM concentration) on cardiac myocyte cell viability after 4 hours normoxia followed by 2 hours of simulated reperfusion. Data are expressed in the ratio of vehicle (DMSO) control in percent. *$p<0.05$ vs. Vehicle, n=5-6 (One-way ANOVA followed by Dunnett post hoc test).
